# A Web-Based Virtual Environment Behavioral Intervention as Cardiovascular Disease and Metabolic Disease Prevention Education in Persons With HIV: Evaluation of the LEARN Randomized Controlled Trial

**DOI:** 10.2196/91145

**Published:** 2026-06-15

**Authors:** S Raquel Ramos, Lindsie Boerger, Arjee Javellana Restar, Manali Phadke, Sangchoon Jeon, Constance Johnson, Gail Melkus, Trace Kershaw, Harmony Reynolds, Allison Vorderstrasse

**Affiliations:** 1 School of Nursing Yale University Orange, CT United States; 2 Department of Social and Behavioral Sciences School of Public Health Yale University New Haven, CT United States; 3 Center for Interdisciplinary Research of AIDS New Haven, CT United States; 4 Yale Center for Clinical Investigation New Haven, CT United States; 5 Yale Center for Analytical Sciences Yale School of Public Health New Haven, CT United States; 6 Cizik School of Nursing UT Health Houston Houston, TX United States; 7 McWilliams School of Biomedical Informatics UT Health Houston Houston, TX United States; 8 Rory Myers College of Nursing New York University New York, NY United States; 9 Department of Medicine Grossman School of Medicine New York University New York, NY United States; 10 Cardiovascular Clinical Research Center NYU Langone Health New York, NY United States; 11 Elaine Marieb College of Nursing University of Massachusetts Amherst Amherst, MA United States

**Keywords:** virtual environment, gamification, preventative cardiology, chronic illness, HIV, CVD, metabolic disease, AR, VR, health education, lifestyle, RCT, minority groups

## Abstract

**Background:**

Persons living with HIV have an increased risk of cardiovascular and metabolic diseases compared with HIV-seronegative individuals.

**Objective:**

This study aims to assess the feasibility and acceptability of a virtual environment (VE) for cardiovascular disease and metabolic disease prevention education among men living with HIV through a pilot waitlist-control randomized trial.

**Methods:**

In phase 1, we conducted interviews and VE beta testing with 25 individuals. In phase 2, reported here, we conducted a waitlist-controlled trial using permuted block randomization. Participants were allocated to either the VE intervention (n=40) or the waitlist control arm (n=38). The primary outcomes were feasibility (defined by recruitment and retention metrics) and acceptability (defined by levels of engagement) of the VE. We also examined preliminary effect sizes across several cardiovascular health–related indicators.

**Results:**

Participants had a mean age of 41.8 (SD 10.4) years and had been living with HIV for a mean of 15.9 (SD 9.7) years. Of the 56 total participants, 35 (63%) were Black and 15 (27%) were Hispanic. A total of 14 out of 31 (45%) of participants with baseline data in the intervention arm engaged with the VE, with an average session duration of 110 minutes. The most highly engaged content modules were nutrition (146 engagements), oral health (138 engagements), fitness tips and videos (82 engagements), and relaxation techniques (75 engagements). Retention was 48 out of 78 (61%) at the final assessment, which, to our knowledge, is higher than the typical retention rates reported in remote digital health and behavioral intervention research. Preliminary efficacy analyses of changes at 3 and 6 months revealed medium effect sizes favoring the intervention for key nutritional outcomes (vegetable consumption: d=0.59-0.66; whole grain intake: d=0.41-0.46) and vigorous physical activity (d=0.38-0.57). Small-to-medium effect sizes were observed for fast food reduction (d=0.37 at 6 months), total physical activity (d=0.37 at 6 months), depressive symptoms (d=0.22 at 3 months), and HIV illness perceptions, including Timeline Acute/Chronic subscale score (d=0.44 at 3 months), Illness Coherence (d=0.33 at 3 months), and Emotional Representations (d=0.23-0.33).

**Conclusions:**

The LEARN (LEveraging A viRtual eNvironment to enhance prevention of HIV-related comorbidities) study demonstrated the feasibility and acceptability of a VE designed for cardiovascular and metabolic disease prevention education among persons living with HIV. High engagement with specific educational content and favorable retention rates highlight the promise of this innovative approach. Preliminary effect sizes suggested positive trends in cardiovascular health indicators and mental well-being, indicating the potential for additional benefits associated with the intervention. These findings support progression to a fully powered randomized controlled trial. Subsequent funding has been awarded based on this work to expand the research in LEARN2. We will integrate lessons learned from this study and focus on HIV-related conditions with shared risk factors to enhance the intervention’s impact. By addressing HIV-related conditions with interrelated risk factors, we aim to provide a more comprehensive intervention to improve the overall health and well-being of individuals living with HIV.

**Trial Registration:**

ClinicalTrials.gov NCT05242952; https://clinicaltrials.gov/ct2/show/NCT05242952

**International Registered Report Identifier (IRRID):**

RR2-10.2196/38348

## Introduction

Cardiovascular disease (CVD) and metabolic disorders pose significant health challenges, particularly among individuals living with HIV. These conditions not only contribute to morbidity and mortality but also complicate HIV management. The incidence of HIV remains highest among Black (26%) and Hispanic (24%) men [1] with incomes at or below the poverty threshold [2]. CVD has emerged as the leading cause of mortality among people with HIV [3-5], primarily because of chronic inflammation associated with the virus, which elevates CVD risk even when viral loads are well controlled [6-9]. Additionally, allostatic load adversely affects the immune, cardiovascular, and metabolic systems over time [10], leading to increased stress responses. These responses elevate cortisol and other inflammatory biomarkers, resulting in physiological disruptions [11] that contribute to cardiometabolic morbidities [12]. Although many CVD risk factors are modifiable, people living with HIV remain at elevated risk because of both shared risk factors with CVD and HIV-specific risk factors.

CVD risk among Black and Hispanic men living with HIV arises from the combined effects of systemic mistreatment, physiological stress responses, and aging with chronic illness. Conceptually referred to as a “cardiovascular conundrum,” Thayer and colleagues [13,14] elucidate how stress responses to systemic mistreatment experienced by Black individuals and other underresourced groups [15] lead to elevated systemic vascular resistance, thereby increasing morbidity and mortality risks associated with CVD. Notably, higher vagally mediated heart rate variability, which is typically associated with lower CVD risk, can be negatively affected by consistent exposure to interpersonal conflict, leading to reduced heart rate variability over time [14,16,17]. Compared with the general US population, individuals living with HIV have a CVD risk that is 1.5-2 times higher, with this risk increasing with age [5,15,18]. By 2030, nearly 80% of individuals living with HIV will be 50 years or older and have 1 or more chronic conditions [19], highlighting the urgent need to address cardiovascular risk in this population.

Innovative approaches to lowering CVD risk among men living with HIV, including behavioral prevention, education, and technological strategies, are essential to address pressing health issues in this population. In 2024, a study by the Pew Research Center [20] indicated that the digital divide has significantly narrowed, with up to 90% of adults aged 65 years and older and 91% of individuals living in poverty reporting internet use. A 2022 nationally representative survey conducted by AARP found that 45% of individuals aged 50 years and older engage in video gaming to maintain mental sharpness, reduce stress, entertain themselves, and solve problems [21]. Research using serious games has been applied to social connection and education after stroke [22], diabetes self-management [23], virtual environment (VE)–based exercise among persons with diabetes [24], self-management and lifestyle changes among individuals with bipolar disorder [25], and cardiac rehabilitation [26]. Although core health promotion principles apply across populations, men living with HIV face unique challenges that may benefit from VE-based interventions, particularly because they experience intersecting stigmas related to HIV status, sexual minority identity, and race/ethnicity that may reduce engagement with traditional health care settings [13,14]. VEs offer distinct advantages for CVD prevention education in this population, including anonymity that may reduce stigma-related barriers to engagement, flexibility for self-paced learning that accommodates varying health literacy levels, and immersive gamification elements that can enhance motivation and sustained engagement with health content [22-26]. Taken together, these findings highlight the potential for technology-driven VE-based behavioral interventions to serve as effective tools for health education and engagement, particularly among populations at elevated risk, such as men living with HIV.

The Second Life Impacts Diabetes Education & Self-Management (SLIDES) study [27] and the Learning in a Virtual Environment (LIVE) platform [28] were disease-agnostic VEs previously tested for diabetes self-management. Our team modified the LIVE platform by shifting its focus and content toward CVD and metabolic disease prevention education for sexual minority men living with HIV, entitled “The LEARN Study” (“LEveraging A viRtual eNvironment to enhance prevention of HIV-related comorbidities”). Briefly, the LEARN study incorporated gamification and educational quests to enhance participant engagement while increasing awareness of cardiovascular health, risk factors, and effective prevention strategies. The platform was personalized for cultural salience and content through formative work described elsewhere [28]. The intervention was informed by Social Cognitive Theory, emphasizing self-efficacy and observational learning [29], and Self-Determination Theory, emphasizing autonomous motivation [30]. VE components, including gamification, self-directed exploration, and personalized avatars, were designed to enhance self-efficacy, autonomous motivation, and knowledge acquisition, which were hypothesized to influence behavioral and health outcomes. The aims of the LEARN study were to (1) evaluate the feasibility and acceptability of this pilot intervention among persons living with HIV and (2) examine the preliminary efficacy of the intervention across health indicators.

## Methods

### Design

Our study evaluation is reported in accordance with the CONSORT (Consolidated Standards of Reporting Trials)-EHEALTH guidelines [31].

To the team’s knowledge, the LEARN Study was the first waitlist control randomized trial to test the feasibility and acceptability of a VE for CVD and metabolic disease prevention education among men living with HIV [32,33]. The waitlist design was advantageous in mitigating ethical concerns for the control group because all participants ultimately received the intervention, particularly in pilot and efficacy trials for which no standard-of-care treatment was available [34]. Before the pilot intervention, our team conducted online-only qualitative interviews with 15 individuals and beta testing of the VE with 10 individuals. Findings related to VE design and personalization that informed the LEARN clinical trial are described elsewhere [32,33,35,36]. Data suggested that participants were enthusiastic about the potential of a VE to address health risks associated with HIV and desired greater representation in avatar customization options. In this study, enrolled participants completed the pilot trial over a 6-month period based on their random assignment, with outcomes measured at 3 time points: baseline, 3 months, and 6 months. The 6-month study duration was selected based on evidence that behavioral lifestyle changes typically require 3-6 months to become established and that longer follow-up periods allow assessment of behavior maintenance beyond initial adoption. This time frame aligns with prior VE intervention studies and permits detection of both short-term (3-month) and sustained (6-month) effects [32,33,35,36].

### Ethical Considerations

All study procedures were approved by the Yale Human Research Protection Program (institutional review board approval number 2000031403). This approval covered all study phases. The protocol was registered on ClinicalTrials.gov (NCT05242952). Informed consent was obtained verbally by the study coordinator during the screening phone call, and participants provided consent before enrollment and data collection. To protect privacy and confidentiality, participants created pseudonyms for their VE avatars, and no personally identifiable information was displayed within the VE. All data were stored in REDCap (Vanderbilt University), a Health Insurance Portability and Accountability Act (HIPAA)-compliant electronic data capture system with role-based access controls. Participants received compensation of US $30 for each completed assessment (baseline, 3 months, and 6 months), for a maximum total of US $90.

### Participants

Eligibility criteria were (1) age 30 years or older, (2) male sex, (3) HIV-seropositive status, (4) ability to participate, and (5) no medical history of serious complications such as heart attack, stroke, or cognitive impairment. Ability to participate was operationalized as (1) having reliable internet access, (2) access to a computer capable of running the VE platform, (3) self-reported ability to read and understand English, and (4) no self-reported visual or motor impairments that would prevent VE navigation. These criteria were assessed during the screening phone call. Additionally, eligibility was not restricted by race/ethnicity; however, recruitment strategies prioritized community partnerships and outreach in settings serving populations with the highest HIV burden.

The rationale for inclusion was grounded in research suggesting that chronic illnesses are increasingly being diagnosed earlier in life, particularly among individuals aged 18-30 years, with the highest prevalence observed among those aged 50 years and older. Data from the Million Hearts study [37] indicate that more than 35% of life-changing cardiac events occur in adults as young as 35 years. Accordingly, our sample age range, beginning at 30 years, was appropriate given the evolving age demographics associated with CVD risk. The focus on men aligns with epidemiologic patterns demonstrating higher HIV incidence among Black and Hispanic men and elevated cardiovascular risk among men aging with HIV [1,2]. The ability to participate was necessary for sustained engagement with a VE that incorporated quests, navigation, and health information. Excluding individuals with prior serious cardiac events or cognitive impairment protected participant safety and avoided potential confounding related to advanced disease or impaired capacity to complete VE activities.

### LEARN Intervention

We conducted the LEARN study by customizing the LIVE disease-agnostic platform [28]. The development process for LIVE is described elsewhere [28] and was previously tested for diabetes self-management through the SLIDES study [27].

**Figure 1:**
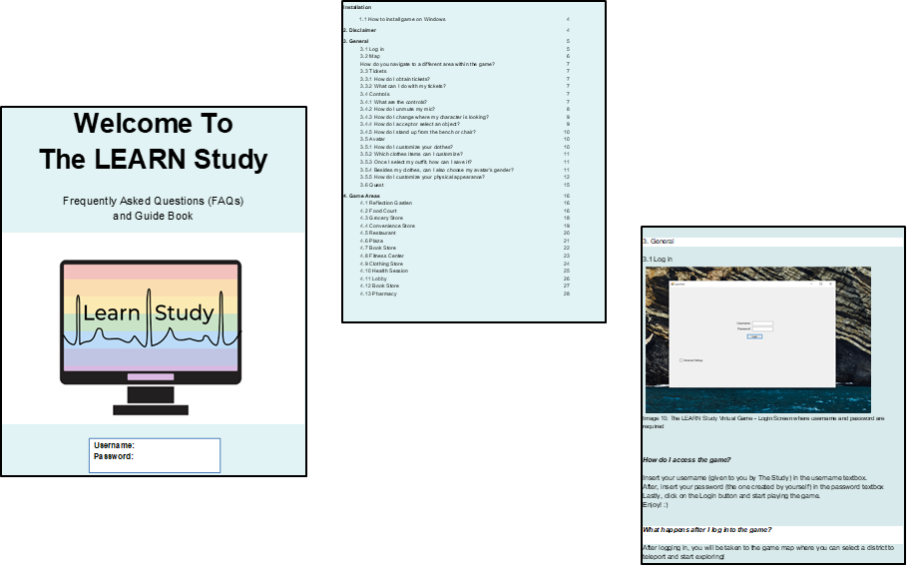
LEARN (LEveraging A viRtual eNvironment to enhance prevention of HIV-related comorbidities) orientation packet.

**Figure 2:**
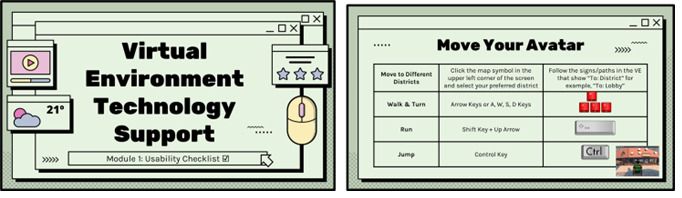
LEARN (LEveraging A viRtual eNvironment to enhance prevention of HIV-related comorbidities) troubleshooting content.

The LEARN intervention included an orientation packet detailing step-by-step login instructions for the VE (Figure 1), describing the VE districts (eg, lobby, food court, plaza, and reflection garden), and outlining the activities available in each area. Its primary purpose was to familiarize participants with the VE’s features and facilitate basic troubleshooting. We also provided a suite of digital support materials. A student research assistant produced a series of brief tutorial videos using Microsoft PowerPoint (each under 6 minutes) covering essential troubleshooting, navigation strategies, and “tips and tricks” for effective engagement within the VE (Figure 2). These orientation videos were distributed via email and posted on the VE’s internal message boards.

**Figure 3:**
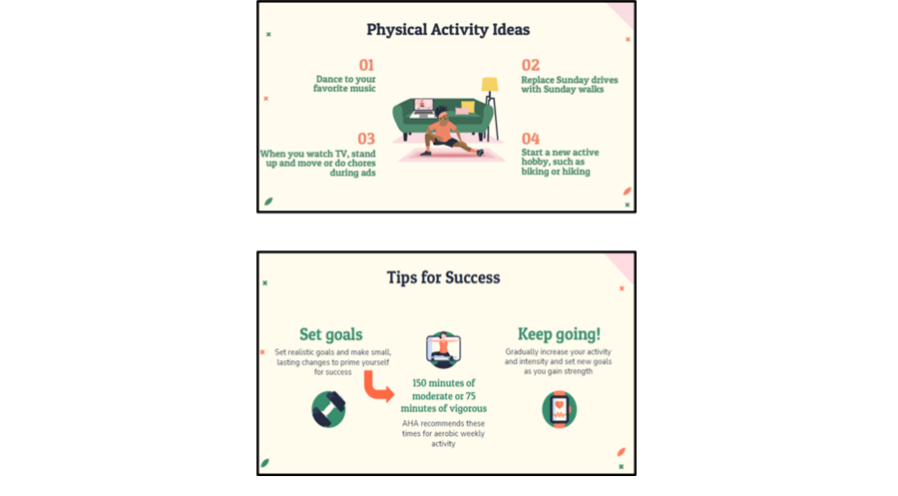
LEARN health video content. LEARN: LEveraging A viRtual eNvironment to enhance prevention of HIV-related comorbidities.

Informed by LEARN qualitative findings [32,33,35,36], the platform was tailored to participants’ preferences and personalized to include a pseudonym for use with their avatar when logged into the VE. Pseudonyms were also used to protect participants’ personally identifiable information. Once onboarded, participants engaged in interactive, self-directed exploration within the VE. They customized their avatars (eg, hair color, skin color, clothing, and footwear) to reflect personal preferences and identity considerations, navigated across multiple VE districts, and encountered targeted health content designed to promote behavioral modification. Within each district, participants were presented with information and images relevant to cardiovascular and metabolic health, such as making dietary choices in the food court or exploring physical activity resources in the plaza, to reinforce key prevention messaging in an immersive context. In parallel, a nurse health educator provided 2 live sessions within the VE and created several videos on topics aligned with the American Heart Association Cardiovascular Health framework (Figure 3) [38]. Recorded versions of these sessions were also posted and emailed to participants as asynchronous reinforcement. Although these resources had limited live integration, they served a critical role in participant onboarding, engagement, and knowledge acquisition.

Engagement with the VE was primarily self-directed; however, participants received (1) monthly email newsletters (not containing CVD-related content) to maintain study contact, (2) email notifications when new VE content was posted, and (3) invitations to 2 live health educator sessions within the VE. These communication strategies supported participant retention (discussed further below) while preserving the self-directed nature of the intervention.

### Recruitment, Screening, and Retention Approaches

We collaborated with the Yale Center for Clinical Investigation to support our clinical trial. A research coordinator with expertise in clinical trial screening, enrollment and consent, data collection, tracking, documentation, and regulatory compliance supported the team throughout the study. We used several recruitment strategies. First, we contacted partnering community-based organizations and distributed recruitment flyers, while program managers informed clients about the study through word of mouth. Second, we implemented a MyChart messaging campaign targeting patients who might be eligible for the research and had agreed to be contacted. Third, the research coordinator contacted patients who had previously expressed interest in clinical trials using a recruitment center call list. Fourth, we partnered with an external investigator and used a listserv of 1800 individuals who had consented to be contacted regarding HIV health research studies. Additionally, the protocol was publicly accessible through registration on ClinicalTrials.gov (NCT05242952).

Between July 2023 and December 2024, individuals were screened for participation. Those who met the eligibility criteria provided informed consent to the research coordinator by phone. Once consented, participants were allocated either to immediate access to the VE or to a waitlist control arm using permuted-block randomization with varying block sizes to ensure allocation concealment and balanced assignment between the intervention and control groups throughout the study period [39]. Additionally, the coordinator verified each participant’s identity through confirmatory phone calls and email confirmations to prevent individuals from enrolling multiple times.

**Figure 4:**
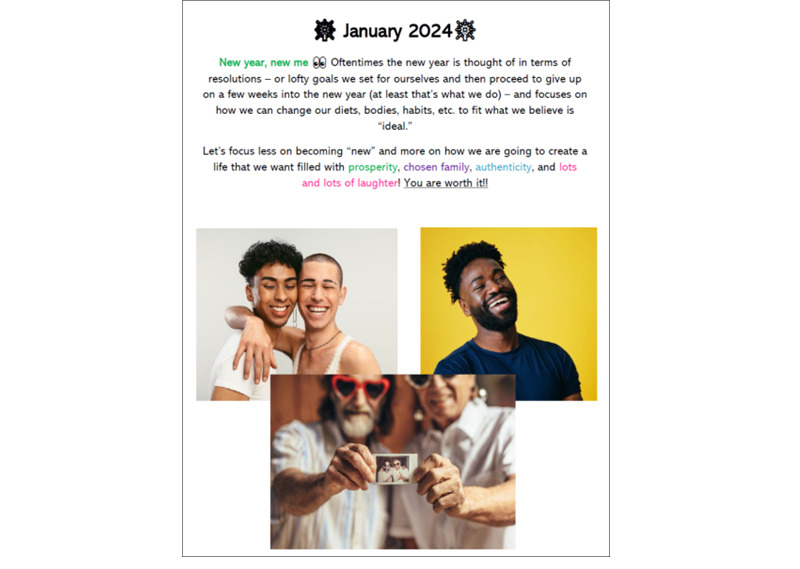
LEARN (LEveraging A viRtual eNvironment to enhance prevention of HIV-related comorbidities) Newsletter.

To enhance retention, the study coordinator maintained regular contact with participants by sending voicemail and email reminders regarding upcoming data collection time points. Monthly newsletters were also distributed via email to support ongoing study communication (Figure 4). These newsletters intentionally excluded content related to CVD or metabolic disease prevention to ensure that retention communications remained distinct from the active intervention and did not influence study outcomes. The primary purpose of the newsletters was to engage participants indirectly by maintaining an open communication channel and acknowledging seasonal occasions and relevant events meaningful to participants.

### Sample Size Calculation

We evaluated the acceptability and feasibility of the VE while obtaining preliminary effect size estimates of its potential impact on several CVD- and HIV-related health indicators. It is important to clarify that this study was not intended to serve as a definitive test of intervention efficacy. Previous research indicated that feasibility studies with a sample size of at least 10 participants were adequate to achieve these objectives [40-42]. Following recommendations from these studies, sample size was determined based on the precision of effect size estimation rather than statistical power for hypothesis testing, as the study was not designed as a definitive efficacy trial. With 40 participants per group, we anticipated 95% CIs for standardized mean differences with a half-width of approximately 0.45 SD, providing sufficient precision to inform decisions regarding progression to a fully powered trial.

### Measures

Self-reported survey data were collected through scheduled phone calls with the study coordinator at baseline, 3-month, and 6-month time points. The coordinator entered participant responses into REDCap, which included demographic, behavioral, and health indicators. Survey data collection required approximately 60-90 minutes. Acceptability data were assessed using engagement metrics derived from the VE platform’s metadata. These metadata included participant login records and log file analyses, which are digital records capturing activities within the VE. This information provided insights into login frequency and duration of platform use during the study period and was logged under each participant’s avatar pseudonym.

### Primary Outcomes

The primary outcomes of this study were the feasibility and acceptability of the VE. Feasibility was operationalized through recruitment and retention metrics. Recruitment metrics were assessed by tracking the proportion of individuals who enrolled among those initially contacted. Retention metrics were measured as the percentage of participants who completed the 3- and 6-month follow-up assessments. Acceptability was evaluated through engagement metrics within the VE, quantified by calculating the total time participants engaged with the platform (in minutes). This included the duration spent visiting various districts (eg, grocery, pharmacy, bookstore, and outlet districts) and interacting with health content modules (eg, nutrition, oral health, fitness tips and videos, and relaxation techniques).

### Demographics

Participant demographic characteristics were collected at baseline through self-report questionnaires. Variables assessed included age (continuous, in years), years living with HIV (continuous, in years), race/ethnicity (categorized as Black, Hispanic/Latine, Asian, Middle Eastern, or other), relationship status (categorized as divorced, married, partnered, or single), education level (categorized as did not complete high school, high school graduate or General Educational Development, some college or associate degree, bachelor’s degree, or master’s degree), and employment status (categorized as employed, on disability, self-employed, student, unemployed, or seasonal).

### Health Indicators

Multiple cardiovascular health indicators were assessed at baseline, 3-month, and 6-month follow-ups using self-report measures aligned with the American Heart Association Life’s Essential Eight metrics for cardiovascular health [38]. We assessed self-reported history of hypertension (yes/no) and diabetes (yes/no). Sleep was measured by hours of sleep per night, with adequacy evaluated against the recommended 7-9 hours [43]. BMI was calculated from self-reported height and weight (kg/m^2^) and categorized as underweight (<18.5 kg/m^2^), normal weight (18.5-24.9 kg/m^2^), overweight (25.0-29.9 kg/m^2^), or obese (≥30.0 kg/m^2^) [44]. Nutritional intake was assessed through average daily servings of vegetables, fruit, and whole grains, along with weekly consumption of sweets, with adherence evaluated against recommendations of 3 servings of vegetables and 2.5 servings of fruit daily [45,46]. Physical activity was assessed using the International Physical Activity Questionnaire, which measures self-reported physical activity in minutes per week, including walking, moderate activity, and vigorous activity [47]. Mental health was evaluated using the 9-item Patient Health Questionnaire-9 to assess depressive symptoms, with participants categorized as having mild, moderate, or severe depression [48]. Tobacco and e-cigarette use were also assessed, including smoking status and quit attempts, using the Behavioral Risk Factor Surveillance System Questionnaire [49].

HIV illness perceptions were assessed using the Revised Illness Perception Questionnaire (IPQR), adapted for HIV, which measures cognitive and emotional representations of illness [50]. The IPQR includes an identity subscale assessing symptoms attributed to HIV and 7 belief subscales measured on a 5-point Likert scale: Timeline Acute/Chronic, Timeline Cyclical, Consequences, Personal Control, Treatment Control, Illness Coherence, and Emotional Representations. Higher scores indicate stronger beliefs within each domain. The IPQR has demonstrated good internal consistency (Cronbach α=.71-.87) across diverse patient populations, including persons living with HIV [50].

We also assessed additional health indicators, including the ability to receive medical care when needed (yes/no) and current health insurance coverage (yes/no). Health literacy was measured using the Standard Assessment of Health Literacy [51], which evaluates an individual’s understanding of common medical terms through word recognition and multiple-choice comprehension items. Internalized homophobia was assessed using the Internalized Homophobia Scale [52,53], a validated instrument that measures the extent to which individuals have internalized negative societal attitudes related to identifying as a sexual minority. The scale includes items assessing personal discomfort with one’s sexual orientation; desire to change sexual orientation; and negative feelings about being gay, lesbian, or bisexual. Higher scores indicate greater levels of internalized homophobia. Sexual orientation concealment was measured using the Sexual Orientation Concealment Scale [54], which evaluates concealment behaviors across various settings, including the workplace, family, health care settings, and social situations. Items assess both active concealment strategies (eg, avoiding disclosure and presenting as heterosexual) and vigilance related to identity management. Higher scores indicate greater concealment of sexual orientation.

### Statistical Analysis

Outcome assessor blinding was not feasible because data collection was conducted by the study coordinator through phone-administered surveys, which required knowledge of participant allocation to coordinate VE access and provide troubleshooting support for intervention participants. To minimize bias, the principal investigator (PI) and study coordinator were excluded from data analysis, which was conducted independently by the biostatistician.

This analysis focused on the 59 participants who completed baseline assessments (31 intervention and 28 control). Means and standard deviations were used to describe continuous variables, whereas frequencies (n) and percentages (%) were used to describe categorical variables, as appropriate. Associations between outcome and demographic variables by group at baseline were assessed using Student *t* tests for continuous outcome variables and chi-square tests of independence for categorical variables. For preliminary efficacy testing, health indicators were collected and analyzed longitudinally across 3 time points (baseline, 3 months, and 6 months) and compared between the intervention and waitlist control groups. A strict intention-to-treat approach was not applied. Rather, participants with at least one data point were included in the longitudinal analyses using linear mixed-effects models, which accommodate missing data under the assumption that data are missing at random. Linear mixed-effects models with restricted maximum likelihood estimation were used to examine differences in each health indicator between the intervention and control groups over time. The model included fixed effects for randomization group, visit time, and the group × time interaction, as well as age, years living with HIV, income category, and education category as covariates. A random intercept was included to account for within-subject correlation across repeated measurements. Type III tests of fixed effects were used to evaluate the significance of each term in the model. Differences in least squares means between the intervention and control groups were estimated at each time point and are presented as effect size estimates with 95% CIs. Statistical significance was set at α=.05. All analyses were conducted using SAS version 9.4 (SAS Institute Inc) [55].

Analyses of engagement and acceptability metrics (VE login sessions, content module access, and session duration) applied only to the intervention arm because control participants did not have access to the VE during the waitlist period. Effect size estimates for health indicators represent between-group differences (intervention vs control) at each time point.

## Results

### Participant Demographics

**Figure 5:**
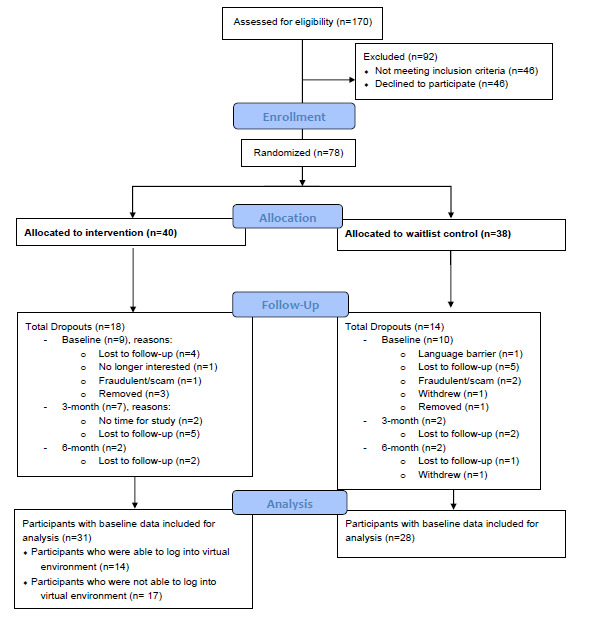
CONSORT (Consolidated Standards of Reporting Trials)-EHEALTH flowchart.

Table 1 summarizes participant demographic characteristics. The mean age of participants was 41.8 (SD 10.4) years. The waitlist control group had a mean age of 42.6 (SD 11.2) years, whereas the intervention group had a mean age of 41.1 (SD 9.7) years. Participants had been living with HIV for a mean of 15.9 (SD 9.3) years. The racial/ethnic composition (N=56) was as follows: 35 (63%) participants were Black, 15 (27%) were Hispanic/Latin American, 1 (2%) was Asian, and 1 (2%) was Middle Eastern. Overall, 36 out of 58 (62%) participants were single and 13 out of 58 (22%) were married. Participants reported a range of educational backgrounds, with the largest proportion having completed some college (17/58, 29%), followed by those with bachelor’s degrees (14/58, 24.1%) and master’s degrees (14/58, 24%). Employment status varied, with 28 out of 58 (48%) employed and 21 out of 58 (36%) either unemployed or working seasonally (Figure 5).

**Table 1:** LEARN^a^ sample demographic characteristics (N=59).^b^

Characteristics			Total, n (%)^c^		Waitlist control (n=28)^c^		Intervention (n=31)^c^		*P* value^d^			
Age, mean (SD)			41.8 (10.4)		42.6 (11.2)		41.1 (9.7)		.58			
Years living with HIV, mean (SD)			15.9 (9.3)		18.1 (11.1)		13.8 (6.7)		.08			
**Race/ethnicity, n (%)**										.72		
	Asian	1 (2)		0 (0)		1 (4)						
	Black or African American	35 (63)		18 (64)		17 (61)						
	Hispanic or Latin American	15 (27)		7 (25)		8 (29)						
	Middle Eastern	1 (2)		0 (0)		1 (4)						
	Multiple races/mixed races	4 (7)		3 (11)		1 (4)						
	White	0 (0)		0 (0)		0 (0)						
**Relationship status, n (%)**										.42		
	Divorced	1 (2)		1 (4)		0 (0)						
	Married	13 (22)		4 (14)		9 (30)						
	Partnered	8 (14)		4 (14)		4 (13)						
	Single	36 (62)		19 (68)		17 (57)						
**Education** **, n (%)**										.32		
	Did not complete high school	2 (3)		2 (7)		0 (0)						
	High school graduate or General Educational Development	11 (19)		5 (18)		6 (20)						
	Some college or associate’s degree	17 (29)		9 (32)		8 (27)						
	Bachelor’s degree	14 (24)		4 (14)		10 (33)						
	Master’s degree	14 (24)		8 (29)		6 (20)						
**Employment status** **, n (%)**										.49		
	Employed	28 (48)		12 (43)		16 (53)						
	On disability	5 (9)		4 (14)		1 (3)						
	Self-employed	1 (2)		1 (4)		0 (0)						
	Student	3 (5)		1 (4)		2 (7)						
	Unemployed or seasonal	21 (36)		10 (36)		11 (37)						

^a^LEARN: LEveraging A viRtual eNvironment to enhance prevention of HIV-related comorbidities.

^b^Summed counts may differ from the total sample because of missing data.

^c^Column percentages.

^d^Chi-square test if categorical or *t* test for continuous variables.

### Primary Outcomes

#### Feasibility

CONSORT-EHEALTH (Multimedia Appendix 1) provides a summary of the feasibility results. A total of 170 individuals were screened and assessed for eligibility, of whom 92 were excluded (n=46 did not meet the inclusion criteria and n=46 declined to participate). In the randomized controlled trial (RCT), 78 of the targeted 80 (98%) participants were enrolled, demonstrating successful recruitment. Of these 78 participants, 40 were assigned to the intervention group and 38 to the waitlist control group.

The final analytic sample size accounted for participant attrition. A total of 59 participants (n=31 in the intervention group and n=28 in the waitlist control group) were included in the final analytic sample following attrition that occurred primarily before baseline data collection, during which 19 (24%) participants dropped out. An additional 8 (10%) participants withdrew before the second assessment (at 3 months), and 3 (4%) participants withdrew before the third assessment (at 6 months). At study completion, 26 participants remained in the waitlist control group and 22 remained in the intervention group. Reasons for dropout across all time points included loss to follow-up (n=19), language barriers (n=1), lack of continued interest in the study (n=1), insufficient time to complete the study (n=2), fraudulent or attempted multiple enrollments (n=3), participant withdrawal (n=2), and removal from the study (n=4). Following allocation, approximately half of the participants in the immediate intervention arm (n=14, 45%) actively engaged with content in the VE. Within the intervention arm, 14 (45%) participants logged into the VE at least once.

#### Acceptability

Table 2 summarizes the acceptability results. Participants logged a total of 110 sessions, with an average session duration of 1 hour and 50 minutes. Quests were accessed 14 times, with the most frequently visited quests located in the fitness district (64.0%), followed by the grocery district (14.0%) and the pharmacy, bookstore, and outlet districts (7.0% each). The most frequently accessed content areas were nutrition (146 engagements), oral health (138 engagements), fitness tips and videos (82 engagements), and relaxation techniques (75 engagements) (Multimedia Appendix 2).

**Table 2:** LEARN^a^ acceptability results.

Acceptability	Total
Total sessions logged, n	110
Average session duration (minutes)	110
Distinct educational quests accessed, n	14
Most visited districts	Fitness: 64.0% Grocery: 14.0% Pharmacy: 7.0% Bookstore: 7.0% Outlet: 7.0%
Most engaged modules	Nutrition: k=146 Oral health: k=138 Fitness tips and videos: k=82 Relaxation techniques: k=75 Health and beauty: k=73 Skin care: k=71 Topics on diabetes: k=66 Health supplies: k=62 Medication: k=55 Complications: k=41 Supplements: k=41 Fast food: k=39 Fitness videos: k=37 Snacks: k=30 Chain restaurants: k=21 Prepared foods: k=20 Beverages: k=18 Frozen food: k=17 Groceries: k=17 Produce: k=16 Dairy: k=12 Meat and seafood: k=11 Health education and technology support: k=8 Health living: k=7 Bakery: k=3 Packaged: k=2

^a^LEARN: LEveraging A viRtual eNvironment to enhance prevention of HIV-related comorbidities.

### Health Indicators

#### Overview

Table 3 provides a summary of health indicators at baseline.

**Table 3:** LEARN^a^ sample health indicator characteristics (n=59).^b^

Characteristics				Total, n (%)^c^		Waitlist control (n=28), n (%)^c^		Intervention (n=31), n (%)^c^			*P* value^d^			
**Cardiovascular health (history of hypertension or diabetes)**												.54		
	No			42 (71)		21 (75)		21 (68)						
	Yes			17 (29)		7 (25)		10 (32)						
**Sleep (met recommended hours/night)**												.36		
	No, less than 4 hours			20 (34)		12 (43)		8 (26)						
	Somewhat, between 6 and 7 hours			10 (17)		5 (18)		5 (16)						
	Yes, 7-9 hours			29 (49)		11 (39)		18 (58)						
**BMI (kg/m^2^)**											.11			
	Underweight (<18.5)			0 (0)		0 (0.0)		0 (0)						
	Normal weight (18.5-24.9)			23 (43)		13 (48)		10 (37)						
	Overweight (25.0-29.9)			21 (39)		12 (44)		9 (33)						
	Overweight/obese (≥30.0)			10 (19)		2 (7)		8 (30)						
**Nutrition intake, mean (SD)**														
	Vegetable servings			6.4 (4.4)		6.5 (4.7)		6.3 (4.2)			.89			
	Fruit servings			6.6 (6.8)		7.0 (8.7)		6.3 (4.8)			.70			
	Whole grain servings			4.2 (5.8)		3.4 (3.1)		5.0 (7.4)			.29			
	Sweet servings			3.8 (3.4)		3.7 (3.4)		4.0 (3.4)			.71			
	Fast food meals			1.9 (2.1)		1.6 (2.0)		2.1 (2.1)			.38			
	Red meat			4.2 (4.1)		5.9 (4.9)		2.7 (2.6)			.004**			
**Physical activity (** **International Physical Activity Questionnaire** **), mean (SD)**														
	Minutes/week			3725.9 (4151.3)		3601.1 (4346.5)		3869.2 (3992.7)			.81			
	Walking (minutes)			289.5 (344.7)		331.1 (361.5)		251.9 (330.2)			.38			
	Moderate activity (minutes)			288.6 (363.9)		302.6 (371.3)		276.0 (362.7)			.78			
	Vigorous activity (minutes)			199.7 (322.1)		189.9 (285.0)		208.2 (355.8)			.83			
**Mental health, mean (SD)**											.51			
	9-item Patient Health Questionnaire score			5.7 (6.0)		6.3 (6.2)		5.2 (5.9)						
**Smoking status: tobacco or e-cigarette use, n (%)**											.67			
	Current smoker			17 (29)		10 (36)		7 (23)						
	Quit 1 to 5 years ago			2 (3)		1 (4)		1 (3)						
	Quit ≥5 years ago			9 (15)		5 (18)		4 (13)						
	Quit <1 year ago or currently using inhaled nicotine delivery			4 (7)		2 (7)		2 (6)						
	Never smoker			27 (46)		10 (36)		17 (55)						
**HIV Illness perceptions (** **Revised Illness Perception Questionnaire** **), mean (SD)**														
	Timeline Acute Chronic subscale			7.5 (2.1)		6.9 (1.8)		8.0 (2.3)			.15			
	Timeline Cyclical subscale			14.6 (3.8)		14.0 (3.9)		15.2 (3.7)			.26			
	Consequences subscale			15.0 (3.6)		14.8 (3.8)		15.2 (3.4)			.63			
	Personal Control subscale			10.5 (3.5)		9.5 (3.53)		11.1 (2.7)			.02*			
	Treatment Control subscale			10.01 (3.0)		11.1 (2.7)		9.1 (2.9)			.007**			
	Illness Coherence subscale			10.2 (3.8)		10.9 (3.9)		9.5 (3.6)			.18			
	Emotional Representations subscale			20.4 (6.4)		18.8 (6.7)		21.9 (5.9)			.07			
	Number of Symptoms			4.51 (8.8)		5.5 (10.3)		3.7 (7.2)			.43			
**Other health indicators**														
	**Receive medical care when needed, n (%)**										.15			
		No	10 (17)		3 (11)		7 (23)							
		Yes	49 (83)		25 (89)		24 (77)							
	**Health insurance coverage, n (%)**										.80			
		No	4 (7)		2 (7)		2 (6)							
		Yes	54 (93)		25 (93)		29 (94)							
	**Health literacy, n (%)**										.71			
		Low	8 (14)		3 (11)		5 (16)							
		High	51 (86)		25 (89)		26 (84)							
	Internalized homophobia, mean (SD)			17.6 (7.1)		18.5 (7.5)		16.7 (6.9)			.33			
	Sexual orientation concealment, mean (SD)			1.3 (0.5)		1.2 (0.3)		1.3 (0.6)			.65			

^a^LEARN: LEveraging A viRtual eNvironment to enhance prevention of HIV-related comorbidities.

^b^Summed counts may differ from the total sample because of missing data.

^c^Column percentages.

^d^Chi-square test if categorical or *t* test for continuous variables.

#### Cardiovascular Health

Overall, 17 out of 59 (29%) participants reported a history of hypertension or diabetes. A total of 7 (25%) out of 28 participants in the control group reported cardiovascular conditions, compared with 10 out of 31 (32%) in the intervention group. Conversely, 42 (71%) in the total sample reported no history of these conditions (21/28, 75%, in the control group vs 21/31, 68%, in the intervention group).

#### Sleep

Nearly half of the participants (29/59, 49%) met the recommended 7-9 hours of sleep per night. However, 10 (17%) reported sleeping 6-7 hours per night, and 20 (34%) reported inadequate sleep, with some individuals sleeping fewer than 4 hours per night. These findings suggest a need for improved sleep hygiene among participants who did not meet the recommended guidelines.

#### BMI

Overall, 23 out of 54 (43%) participants were classified as normal weight, 21 out of 54 (39%) as overweight, and 10 out of 54 (19%) as obese. A higher proportion of participants in the waitlist control group were classified as normal weight (13/27, 48%) or overweight (12/27, 44%) compared with participants in the intervention group (10/27, 37%, and 9/27, 33%, respectively). By contrast, a higher proportion of intervention participants were classified as obese (8/27, 30%) compared with control participants (2/27, 7%).

#### Nutritional Intake

Vegetable servings averaged 6.4 (SD 4.4) overall, with similar consumption between the control (mean 6.5, SD 4.7) and intervention (mean 6.3, SD 4.2) groups. Fruit servings averaged 6.6 (SD 6.8) overall, with control participants consuming slightly more (mean 7.0, SD 8.7) than intervention participants (mean 6.3, SD 4.8). Whole grain servings averaged 4.2 (SD 5.8) overall, with control participants consuming fewer servings (mean 3.4, SD 3.1) than intervention participants (mean 5.0, SD 7.4). Sweet servings averaged 3.8 (SD 3.4) overall, with similar consumption between the control (mean 3.7, SD 3.4) and intervention (mean 4.0, SD 3.4) groups. Fast-food meals averaged 1.9 (SD 2.1) per week overall, with control participants consuming slightly fewer meals (mean 1.6, SD 2.0) than intervention participants (mean 2.1, SD 2.1). Red meat servings differed significantly between groups, with control participants consuming a mean of 5.9 (SD 4.9) servings compared with 2.7 (SD 2.6) servings among intervention participants.

#### Physical Activity

Physical activity was assessed using the International Physical Activity Questionnaire. For MET (metabolic equivalent of task)-minutes per week, the mean was 3725.9 (SD 4151.3) for the total sample, indicating moderate-to-high overall physical activity levels. Control participants averaged 3601.1 (SD 4346.5) MET-minutes per week, compared with 3869.2 (SD 3992.7) MET-minutes per week in the intervention group. For minutes spent walking, participants reported a mean of 289.5 (SD 344.7) minutes per week. Control participants averaged 331.1 (SD 361.5) minutes, compared with 251.9 (SD 330.2) minutes in the intervention group. Moderate-intensity physical activity averaged 288.6 (SD 363.9) minutes per week, with control participants reporting 302.6 (SD 371.3) minutes compared with 276.0 (SD 362.7) minutes in the intervention group. Vigorous-intensity physical activity averaged 199.7 (SD 322.1) minutes per week, with control participants reporting 189.9 (SD 285.0) minutes compared with 208.2 (SD 355.8) minutes in the intervention group.

#### Mental Health

The mean 9-item Patient Health Questionnaire score was 5.7 (SD 6.0) for the total sample, indicating mild depressive symptoms on average. Scores were similar between groups, with control participants averaging 6.3 (SD 6.2) and intervention participants averaging 5.2 (SD 5.9), with no significant between-group difference.

#### Tobacco or e-Cigarette

Smoking status was assessed across 3 categories. More than one-quarter of participants (17/59, 29%) were current smokers, 2 (3%) had quit smoking within the past 1-5 years, and 9 (15%) had quit smoking 5 or more years ago. Current smoking rates differed between groups, with 10 out of 28 (36%) control participants reporting current smoking compared with 7 out of 31 (23%) intervention group participants.

#### HIV Illness Perceptions via IPQR Subscales

Baseline illness perceptions are presented in Table 4. Most IPQR subscale scores were similar between groups. However, 2 subscales demonstrated significant between-group differences: intervention participants reported higher Personal Control scores (mean 11.1, SD 2.7 vs mean 9.5, SD 3.5; *P*=.02), whereas control participants reported higher Treatment Control beliefs (mean 11.1, SD 2.7 vs mean 9.1, SD 2.9; *P*=.007). Emotional Representations scores trended higher in the intervention group, although this difference was not statistically significant.

**Table 4:** LEARN^a^ preliminary efficacy results.^b^

Intervention vs control			Delta effect size at 3 months (from baseline)	*t* test (*df*)	*P* value	Delta effect size at 6 months (from baseline)	*t* test (*df*)	*P* value
**Cardiovascular health**								
		BMI	0.03 (–)	0.57	.82	–0.03 (+)	0.61	.83
**Nutrition intake**								
		Vegetable servings	0.59 (+)^c^	1.67	.11	0.66 (+)^c^	1.72	.09
		Fruit servings	–0.25 (–)^d^	1.54	.26	0.01 (+)	1.57	.96
		Sweets servings	–0.65 (+)^c^	2.18	.31	0.01 (–)	2.25	.98
		Whole grains servings	0.41 (+)^c^	1.94	.22	0.46 (+)^c^	2.00	.19
		Red meat servings	0.17 (–)	2.13	.74	–0.04 (+)	2.19	.94
		Fast food meals	0.17 (–)	1.15	.76	–0.37 (+)^d^	1.18	.52
**Physical activity (** **International Physical Activity Questionnaire** **)**								
		Met minutes/week	0.17 (+)	1186.22	.54	0.37 (+)^d^	1216.34	.21
		Walking (minutes)	–0.19 (–)	109.06	.55	0.15 (+)	111.71	.65
		Moderate activity (minutes)	0.01 (+)	105.80	.98	–0.06 (–)	108.32	.84
		Vigorous activity (minutes)	0.38 (+)^d^	102.40	.23	0.57 (+)^c^	104.98	.08
**Mental health**								
	9-item Patient Health Questionnaire total score		0.22 (+)^d^	1.24	.29	–0.05 (–)	1.28	.81
**HIV illness perceptions (** **Revised Illness Perception Questionnaire** **)**								
	Timeline Acute Chronic subscale		0.44 (–)^c^	0.66	.17	0.03 (–)	0.69	.94
	Timeline Cyclical subscale		0.02 (–)	0.77	.93	0.09 (–)	0.81	.69
	Consequences subscale		0.30 (–)^c^	0.93	.25	0.02 (–)	0.97	.95
	Personal Control subscale		0.13 (+)	0.83	.60	–0.08 (–)	0.87	.75
	Treatment Control subscale		0.13 (+)	0.71	.59	–0.36 (–)^c^	0.74	.15
	Illness Coherence subscale		–0.33 (+)^d^	0.88	.16	–0.11 (+)	0.92	.67
	Emotional Representations subscale		0.33 (+)^d^	1.12	.07	0.23 (+)^d^	0.18	.22
	Number of Symptoms		0.11 (–)	2.62	.70	0.03 (–)	2.74	.93
**Other mechanisms**								
	Internalized Homophobia Total score		–0.03 (+)	1.33	.88	–0.08 (+)	1.36	.68
	Sexual Orientation Concealment Scale		0.25 (–)^d^	0.17	.46	0.03 (–)	0.18	.92

^a^LEARN: LEveraging A viRtual eNvironment to enhance prevention of HIV-related comorbidities.

^b^“+” indicates toward the hypothesized direction; “–” indicates away from the hypothesized direction.

^c^Medium effect size (ie, 0.40-0.69).

^d^Small effect size (ie, 0.20-0.39).

#### Additional Health Indicators

Most participants (49/59, 83%) reported receiving medical care when needed, with a higher proportion of control participants reporting access (25/28, 89%) compared with intervention participants (24/31, 77%). Most participants (54/58, 93%) had health insurance coverage, with 25 out of 27 (93%) control participants and 29 out of 31 (94%) intervention participants reporting coverage. A majority participants demonstrated high health literacy (51/59, 86%), with comparable rates between groups (25/28, 89%, control participants and 26/31, 84%, intervention participants demonstrated high health literacy). The mean Internalized Homophobia Scale total score was 17.6 (SD 7.1) for the total sample. Control participants reported slightly higher scores (mean 18.5, SD 7.5) than intervention participants (mean 16.7, SD 6.9). Participants reported low levels of sexual orientation concealment, with an overall mean score of 1.3 (SD 0.5). This low score indicates that participants, regardless of group assignment, generally did not conceal their sexual orientation. Scores were nearly identical between the control (mean 1.2, SD 0.3) and intervention (mean 1.3, SD 0.6) groups, suggesting similar levels of openness regarding sexual orientation across groups.

### Intervention Effect Size

#### Overview

Table 4 presents preliminary efficacy results across the health indicators. Below are the effect sizes comparing 3 months with baseline and 6 months with baseline.

#### Cardiovascular Health

BMI demonstrated minimal change across time points, moving away from the hypothesized direction at 3 months (0.03) and toward the hypothesized direction at 6 months (−0.03).

#### Nutritional Intake

Vegetable servings showed consistent improvement, with medium effect sizes of 0.59 at 3 months and 0.66 at 6 months, both in the hypothesized direction. Fruit servings showed mixed results, moving away from the hypothesized direction at 3 months with a small effect size (−0.25) but returning toward the hypothesized direction at 6 months (0.01). Sweets consumption showed a notable reduction at 3 months, with a medium effect size (−0.65) in the hypothesized direction; however, this effect was not sustained at 6 months (0.01, away from the hypothesized direction). Similarly, whole grain servings improved with medium effect sizes at both 3 months (0.41) and 6 months (0.46), each in the hypothesized direction. Red meat consumption moved away from the hypothesized direction at 3 months (0.17) but returned toward the hypothesized direction at 6 months (−0.04). Fast-food consumption similarly moved away from the hypothesized direction at 3 months (0.17) but improved by 6 months, with a small effect size (−0.37) in the hypothesized direction.

#### Physical Activity via International Physical Activity Questionnaire

MET-minutes per week showed progressive improvement, with an effect size of 0.17 at 3 months and a small effect size of 0.37 at 6 months, both in the hypothesized direction. Walking minutes moved away from the hypothesized direction at 3 months (−0.19) but improved toward the hypothesized direction by 6 months (0.15). Moderate-intensity activity minutes showed minimal change at both time points (0.01 at 3 months, in the hypothesized direction, and −0.06 at 6 months, away from the hypothesized direction). Vigorous-intensity activity minutes demonstrated the largest improvements, with a small effect size at 3 months (0.38) and a medium effect size at 6 months (0.57), both in the hypothesized direction.

#### Mental Health

Total 9-item Patient Health Questionnaire scores for depression showed a small improvement at 3 months, with an effect size of 0.22 in the hypothesized direction, but moved slightly away from the hypothesized direction at 6 months (−0.05).

#### HIV Illness Perceptions via IPQR

The Timeline Acute/Chronic subscale moved away from the hypothesized direction, with a medium effect size at 3 months (0.44), but moved closer to the hypothesized direction at 6 months (0.03). The Timeline Cyclical subscale moved away from the hypothesized direction at both 3 months (0.02) and 6 months (0.09). The Consequences subscale also moved away from the hypothesized direction, with a small effect size at 3 months (0.30), although this effect was reduced at 6 months (0.02). Personal Control showed improvement at 3 months (0.13, in the hypothesized direction) but moved away from the hypothesized direction at 6 months (−0.08). Treatment Control similarly improved at 3 months (0.13, in the hypothesized direction) but moved away from the hypothesized direction at 6 months, with a small effect size (−0.36). Illness Coherence improved in the hypothesized direction at both 3 months (−0.33, small effect size) and 6 months (−0.11). Emotional Representations improved in the hypothesized direction, with small effect sizes at both 3 months (0.33) and 6 months (0.23). The Number of Symptoms subscale moved away from the hypothesized direction at both 3 months (0.11) and 6 months (0.03).

For internalized homophobia, the intervention group demonstrated improvement at both 3 months (−0.03) and 6 months (−0.08), indicating reductions in internalized negative feelings related to sexual orientation over time. These effect sizes suggest that the intervention may have positively influenced participants’ self-perception. By contrast, sexual orientation concealment showed a small effect size at 3 months (0.25), indicating movement away from the hypothesized direction and suggesting increased concealment of sexual orientation. However, this effect size decreased substantially at 6 months (0.03), indicating movement back toward the hypothesized direction and suggesting that concealment levels may have stabilized or improved over time. These trends may have been influenced by the timing of the LEARN study in relation to broader external and societal factors affecting perceptions of sexual orientation.

For the pooled baseline effect size analysis, visit time, age, years since HIV diagnosis, income, and education were included as covariates in the calculation of pooled baseline estimates used to derive effect sizes.

## Discussion

### Principal Findings

The LEARN Study represents the first known effort to assess the feasibility and acceptability of a VE for cardiovascular and metabolic disease prevention education among men living with HIV. The novelty of this approach lies in its customized platform integration, gamification, and interactive educational quests designed to enhance participant engagement and awareness of heart-healthy behavioral and lifestyle choices. Our findings indicate that the intervention was both feasible to implement and acceptable to participants, with engagement patterns comparing favorably with those of similar VE interventions.

The findings of the LEARN RCT demonstrated feasibility, as we enrolled 78 participants, achieving 98% of the targeted sample size (N=80). This outcome is noteworthy given that approximately 25% of RCTs fail to meet recruitment goals, even with extended trial periods [56]. The high enrollment rate reflects the effectiveness of the multiple recruitment strategies used, including community partnerships, digital outreach through MyChart messaging campaigns, research listservs, and targeted communications. Retention presented challenges typically associated with technology-driven longitudinal research, with a final retention rate of 61.5% (48 out of 78) at the 6-month assessment. Although modest, our retention outcome exceeded the typical rates observed in remote digital health initiatives, which peak at approximately 56% [57-59]. Attrition primarily occurred before baseline data collection, with 19 out of 78 (24%) participants withdrawing after providing verbal consent, consistent with research indicating that more than half of discontinuations in digital health studies occur within the first week [60]. To enhance retention, various strategies were implemented, including maintaining regular contact through telephone calls and emails and disseminating monthly newsletters celebrating relevant events such as Pride and paying homage to the legacy activists who paved the way (eg, Marsha P. Johnson, Sylvia Rivera, and countless others) toward visibility, representation, and the right to exist. These communications fostered a sense of community connection between the research team and participants. Positive feedback received through “thank-you” emails and phone communications with the study coordinator suggested that maintaining cultural relevance and community acknowledgment, which are key principles of community-based research [61], may be particularly important for engaging underrepresented groups in longitudinal research.

Of note, there were 1 or 2 instances of individuals attempting to enroll multiple times. The study coordinator employed a rigorous, multistep verification process that included phone screenings, email confirmations, checks for geographic inconsistencies, and IP address verification along with location screening. This approach enabled the detection of discrepancies, such as registrations originating from different continents and participants using virtual private networks to mask their actual locations. The challenge of multiple enrollment attempts has become increasingly prevalent in remote research settings, and no evidence-based strategies currently exist to address it [62,63]. However, our comprehensive approach effectively mitigated this risk, resulting in no duplicate enrollments.

Moreover, our findings indicate that the intervention was acceptable. Specifically, engagement metrics demonstrated participants’ willingness to interact with the educational content, with 110 login sessions averaging nearly 2 hours each and 45% (14 out of 31) of intervention participants accessing the VE at least once. These engagement patterns are consistent with findings from similar VE interventions [22,42]. For instance, the SLIDES study, which used a VE for diabetes self-management, reported that participants logged a mean of 2.5 hours per week throughout the study period [42]. The high level of engagement with specific content modules in the LEARN study, particularly nutrition (146 engagements), oral health (138 engagements), fitness tips and videos (82 engagements), and relaxation techniques (75 engagements), suggests that participants were especially interested in informational content that directly affected their daily health choices.

These engagement patterns align with findings from prior VE interventions targeting chronic disease management. For example, the SLIDES study reported that participants logged a mean of 2.5 hours per week over 6 months for diabetes self-management [23,27,42], while Beauchamp et al [22] achieved 93% retention among stroke survivors despite usability challenges, and Touloudi et al [24] demonstrated high acceptance of VE-based exercise in diabetes. However, the LEARN sample differed meaningfully from those in prior studies, as our participants were younger (mean age 41.8 vs 54-59 years), more racially diverse (35/56, 63%, Black; 15/56, 27% Hispanic/Latin American vs 60%-65% White), predominantly single (36/58, 62%, vs 56.3% married), and exclusively male and living with HIV. These demographic differences highlight the importance of tailoring VE interventions to the specific needs and characteristics of target populations, particularly those historically underrepresented in clinical trials and behavioral intervention research. The demographic composition of our sample reflects both the epidemiology of HIV in the United States and our intentional effort to include populations historically underrepresented in clinical trials.

The exploratory effect sizes observed in this study are consistent with, and in some cases exceed, those reported in comparable digital health interventions. For dietary outcomes, medium effect sizes for vegetable consumption (*d*=0.59 at 3 months to 0.66 at 6 months) and whole grain intake (*d*=0.41 at 3 months to 0.46 at 6 months) compare favorably with meta-analytic findings from technology-based dietary interventions, which typically report small-to-medium effects [64,65]. The sustained improvement at 6 months suggests that the VE format may support durable behavior change, although the attenuation in sweets reduction (*d*=–0.65 at 3 months to 0.01 at 6 months) indicates that some dietary behaviors may require ongoing reinforcement. The improvement in vigorous physical activity (*d*=0.38 at 3 months to *d*=0.57 at 6 months) is noteworthy and comparable to findings from a meta-analysis of physical activity interventions showing larger effects at 6-9 months, although the extent to which these interventions were delivered virtually or digitally was not specified [66]. The gamified, self-directed nature of the VE may have promoted autonomous motivation, which has been proposed to support sustained physical activity adherence within self-determination theory frameworks [67,68]. We also observed that more participants with higher BMI were retained in the intervention group than in the control group. Although it is plausible that the virtual format reduced weight-related stigma, offered an engaging self-directed experience, or was perceived as more personally relevant by this subgroup, the reasons for this differential retention warrant further study. Moreover, although any improvement resulting from an intervention not directly targeting mental health or illness perceptions is notable, the small effect sizes for depressive symptoms (*d*=0.22) and illness perceptions (*d*=0.23 at 3 months and *d*=0.33 at 6 months) observed with this low-intensity, self-directed VE intervention warrant further investigation in a fully powered trial. These preliminary shifts in these domains may represent precursors to sustained behavioral change, although larger samples are needed to confirm these effect size patterns.

### Limitations

The study has several limitations that warrant consideration. First, the sample was limited to men living with HIV, which may affect the generalizability of the findings to other populations and chronic conditions. Second, the exclusion of women and nonbinary individuals further limits generalizability. This focus was intentional given the epidemiological burden of both HIV and CVD among men, particularly Black and Hispanic men. Future research should examine the feasibility and acceptability of VE-based CVD prevention education among women and gender-diverse populations living with HIV. Third, technical challenges associated with the platform due to its age (eg, freezing, audio problems, and installation difficulties) likely constrained engagement, although the study team worked closely with developers to provide ongoing troubleshooting support. Fourth, given the study’s retention rate and the decline in engagement over time, future iterations could address these issues by refining screening and inclusion criteria to identify characteristics associated with participant attrition and by developing a new platform. Future versions should also incorporate additional strategies to sustain participant engagement, such as personalized content, regular updates, and incentives for continued participation. Finally, the exploratory nature of the secondary outcomes means that the study was not powered to detect significant changes among the health indicators examined. Future research should build on these exploratory findings to develop and test more targeted interventions aimed at improving specific health behaviors and outcomes.

### Measures of Rigor for Clinical Trials

The methodological rigor of this study helps balance these limitations with several notable strengths. Intervention fidelity was maintained through a comprehensive approach addressing 5 components: (1) intervention design, (2) study staff training, (3) intervention delivery, (4) participant receipt, and (5) outcome assessment. First, all study staff completed ethical research training, participated in role-playing exercises for participant recruitment and enrollment, and followed a detailed scope-of-work document outlining research-related tasks and responsibilities. Second, participant receipt of the intervention was objectively measured using login frequency, engagement duration, and log file data capturing specific activities within the VE. Third, the study coordinator, trained through the Clinical and Translational Science Award program, ensured consistent participant screening, enrollment, consent, and data collection using standardized scripts. Fourth, a statistician designed the permuted block randomization scheme, and although the PI had access to participant allocation, neither the PI nor the study coordinator participated in the data analysis. This approach was intended to minimize bias in outcome ascertainment [69]. Fifth, data collection and storage were conducted using REDCap, a HIPAA-compliant, validated platform recognized for reliability in clinical research. Sixth, the study’s educational content was grounded in the American Heart Association Life’s Essential 8 framework [38] for cardiovascular health, lending credibility to the prevention messaging delivered through the VE [60]. Finally, the novel approach to cardiovascular and metabolic disease prevention education using gamification and interactive quests distinguishes this intervention from traditional clinical patient education. The high enrollment rate, methodological rigor, and use of validated measures strengthen the validity of the findings. The sample’s demographic composition (ie, Black and Hispanic men living with HIV) addresses a critical gap in clinical trial representation. Engagement metrics demonstrating average sessions of nearly 2 hours further highlight participants’ favorable reception of the VE content, supporting the potential of this approach to reach underserved populations through innovative health education.

### Conclusions

The LEARN Study demonstrated the feasibility and acceptability of a VE designed for cardiovascular and metabolic disease prevention education among persons living with HIV. High engagement with specific educational content and favorable retention rates highlight the promise of this innovative approach. Preliminary effect sizes suggested positive trends in cardiovascular health indicators and mental well-being, indicating the potential for additional benefits associated with the intervention. These findings support progression to a fully powered RCT to evaluate the efficacy of VE-based CVD prevention education. Subsequent funding has been awarded based on this work to expand the research. In the subsequent study, LEARN2, we will integrate lessons learned from this trial and focus on HIV-related conditions with shared risk factors to enhance the intervention’s impact. By addressing HIV-related conditions with interrelated risk factors, we aim to provide a more comprehensive intervention to improve the overall health and well-being of individuals living with HIV.

## Appendix

Multimedia Appendix 1 CONSORT-eHEALTH checklist (V 1.6.1).

Multimedia Appendix 2 LEARN (LEveraging A viRtual eNvironment to enhance prevention of HIV-related comorbidities) acceptability results.

## Abbreviations

CONSORT: Consolidated Standards of Reporting Trials
CVD: cardiovascular disease
HIPAA: Health Insurance Portability and Accountability Act
IPQR: Revised Illness Perception Questionnaire
LEARN: LEveraging A viRtual eNvironment to enhance prevention of HIV-related comorbidities
LIVE: Learning in a Virtual Environment
MET: metabolic equivalent of task
PI: principal investigator
RCT: randomized controlled trial
SLIDES: Second Life Impacts Diabetes Education & Self-Management
VE: virtual environment

## Data Availability

The datasets generated and analyzed during this study are not publicly available because of the sensitive nature of HIV-related health information and the need to protect participant privacy. Deidentified data may be made available from the corresponding author (SRR) upon reasonable request and subject to appropriate data use agreements.
