# Supplementary material for: A Web-Based Virtual Environment Behavioral Intervention as Cardiovascular Disease and Metabolic Disease Prevention Education in Persons With HIV: Evaluation of the LEARN Randomized Controlled Trial
Source: J Med Internet Res. 2026 Jun 15;28:e91145. doi: 10.2196/91145 (PMC13316017; doi:10.2196/91145)
Supplement: Multimedia Appendix 1 [file jmir_v28i1e91145_app1.pdf]

CONSORT-EHEALTH (V 1.6.1) - Submission/Publication Form

The CONSORT-EHEALTH checklist is intended for authors of randomized trials evaluating web-based and Internet-based applications/interventions, including mobile interventions, electronic games (incl multiplayer games), social media, certain telehealth applications, and other interactive and/or networked electronic applications. Some of the items (e.g. all subitems under item 5 - description of the intervention) may also be applicable for other study designs.

The goal of the CONSORT EHEALTH checklist and guideline is to be
a) a guide for reporting for authors of RCTs,
b) to form a basis for appraisal of an ehealth trial (in terms of validity)

CONSORT-EHEALTH items/subitems are MANDATORY reporting items for studies published in the Journal of Medical Internet Research and other journals / scientific societies endorsing the checklist.

Items numbered 1., 2., 3., 4a., 4b etc are original CONSORT or CONSORT-NPT (non-pharmacologic treatment) items.
Items with Roman numerals (i., ii, iii, iv etc.) are CONSORT-EHEALTH extensions/clarifications.

As the CONSORT-EHEALTH checklist is still considered in a formative stage, we would ask that you also RATE ON A SCALE OF 1-5 how important/useful you feel each item is FOR THE PURPOSE OF THE CHECKLIST and reporting guideline (optional).

Mandatory reporting items are marked with a red *.
In the textboxes, either copy & paste the relevant sections from your manuscript into this form - please include any quotes from your manuscript in QUOTATION MARKS,
or answer directly by providing additional information not in the manuscript, or elaborating on why the item was not relevant for this study.

YOUR ANSWERS WILL BE PUBLISHED AS A SUPPLEMENTARY FILE TO YOUR PUBLICATION IN JMIR AND ARE CONSIDERED PART OF YOUR PUBLICATION (IF ACCEPTED).
Please fill in these questions diligently. Information will not be copyedited, so please use proper spelling and grammar, use correct capitalization, and avoid abbreviations.

DO NOT FORGET TO SAVE AS PDF _AND_ CLICK THE SUBMIT BUTTON SO YOUR ANSWERS ARE IN OUR DATABASE !!!

Your response is too large. Try shortening some answers.

Eysenbach G, CONSORT-EHEALTH Group

CONSORT-EHEALTH: Improving and Standardizing Evaluation Reports of Web-based and Mobile Health Interventions
J Med Internet Res 2011;13(4):e126
URL: http://www.jmir.org/2011/4/e126/
doi: 10.2196/jmir.1923
PMID: 22209829

Sign in to Google to save your progress. Learn more

* Indicates required question

Your name *

First Last

S. Raquel Ramos

Primary Affiliation (short), City, Country *

University of Toronto, Toronto, Canada

Yale University, Orange, CT, USA

Your e-mail address *

abc@gmail.com

raquel.ramos@yale.edu

Title of your manuscript *

Provide the (draft) title of your manuscript.

A Web-based Virtual Environment Behavioral Intervention as CVD and Metabolic Disease Prevention Education: Evaluation of the LEARN RCT

Your response is too large. Try shortening some answers.

Name of your App/Software/Intervention *

If there is a short and a long/alternate name, write the short name first and add the long name in brackets.

The LEARN Study

Evaluated Version (if any)

e.g. "V1", "Release 2017-03-01", "Version 2.0.27913"

Your answer

Language(s) *

What language is the intervention/app in? If multiple languages are available, separate by comma (e.g. "English, French")

English

URL of your Intervention Website or App

e.g. a direct link to the mobile app on app in appstore (itunes, Google Play), or URL of the website. If the intervention is a DVD or hardware, you can also link to an Amazon page.

N/A

URL of an image/screenshot (optional)

Your answer

Your response is too large. Try shortening some answers.

Accessibility *

Can an enduser access the intervention presently?

- ○ access is free and open
- ○ access only for special usergroups, not open
- ○ access is open to everyone, but requires payment/subscription/in-app purchases
- ◉ app/intervention no longer accessible
- ○ Other:

Primary Medical Indication/Disease/Condition *

e.g. "Stress", "Diabetes", or define the target group in brackets after the condition, e.g. "Autism (Parents of children with)", "Alzheimers (Informal Caregivers of)"

CVD and Metabolic disease prevention in pers[illegible]

Primary Outcomes measured in trial *

comma-separated list of primary outcomes reported in the trial

Feasibility and Acceptability

Secondary/other outcomes

Are there any other outcomes the intervention is expected to affect?

Cardiovascular health indicators

Your response is too large. Try shortening some answers.

Recommended "Dose" *

What do the instructions for users say on how often the app should be used?

- (x) Approximately Daily
- ( ) Approximately Weekly
- ( ) Approximately Monthly
- ( ) Approximately Yearly
- ( ) "as needed"
- ( ) Other: ____

Approx. Percentage of Users (starters) still using the app as recommended after 3 months *

- ( ) unknown / not evaluated
- ( ) 0-10%
- ( ) 11-20%
- ( ) 21-30%
- ( ) 31-40%
- ( ) 41-50%
- ( ) 51-60%
- (x) 61-70%
- ( ) 71%-80%
- ( ) 81-90%
- ( ) 91-100%

Your response is too large. Try shortening some answers.

Overall, was the app/intervention effective? *

- ( ) yes: all primary outcomes were significantly better in intervention group vs control
- (x) partly: SOME primary outcomes were significantly better in intervention group vs control
- ( ) no statistically significant difference between control and intervention
- ( ) potentially harmful: control was significantly better than intervention in one or more outcomes
- ( ) inconclusive: more research is needed
- ( ) Other:

Article Preparation Status/Stage *

At which stage in your article preparation are you currently (at the time you fill in this form)

- ( ) not submitted yet - in early draft status
- ( ) not submitted yet - in late draft status, just before submission
- (x) submitted to a journal but not reviewed yet
- ( ) submitted to a journal and after receiving initial reviewer comments
- ( ) submitted to a journal and accepted, but not published yet
- ( ) published
- ( ) Other:

Your response is too large. Try shortening some answers.

Journal *

If you already know where you will submit this paper (or if it is already submitted), please provide the journal name (if it is not JMIR, provide the journal name under "other")

- ○ not submitted yet / unclear where I will submit this
- ◉ Journal of Medical Internet Research (JMIR)
- ○ JMIR mHealth and UHealth
- ○ JMIR Serious Games
- ○ JMIR Mental Health
- ○ JMIR Public Health
- ○ JMIR Formative Research
- ○ Other JMIR sister journal
- ○ Other:

Is this a full powered effectiveness trial or a pilot/feasibility trial? *

- ◉ Pilot/feasibility
- ○ Fully powered

Manuscript tracking number *

If this is a JMIR submission, please provide the manuscript tracking number under "other" (The ms tracking number can be found in the submission acknowledgement email, or when you login as author in JMIR. If the paper is already published in JMIR, then the ms tracking number is the four-digit number at the end of the DOI, to be found at the bottom of each published article in JMIR)

- ○ no ms number (yet) / not (yet) submitted to / published in JMIR

Your response is too large. Try shortening some answers.

TITLE AND ABSTRACT

1a) TITLE: Identification as a randomized trial in the title

1a) Does your paper address CONSORT item 1a? *

I.e does the title contain the phrase "Randomized Controlled Trial"? (if not, explain the reason under "other")

- (selected) yes
- ( ) Other:

1a-i) Identify the mode of delivery in the title

Identify the mode of delivery. Preferably use “web-based” and/or “mobile” and/or “electronic game” in the title. Avoid ambiguous terms like “online”, “virtual”, “interactive”. Use “Internet-based” only if Intervention includes non-web-based Internet components (e.g. email), use “computer-based” or “electronic” only if offline products are used. Use “virtual” only in the context of “virtual reality” (3-D worlds). Use “online” only in the context of “online support groups”. Complement or substitute product names with broader terms for the class of products (such as “mobile” or “smart phone” instead of “iphone”), especially if the application runs on different platforms.

	1	2	3	4	5	
subitem not at all important	( )	( )	( )	( )	(selected)	essential

Clear selection

Your response is too large. Try shortening some answers.

Does your paper address subitem 1a-i? *

Copy and paste relevant sections from manuscript title (include quotes in quotation marks "like this" to indicate direct quotes from your manuscript), or elaborate on this item by providing additional information not in the ms, or briefly explain why the item is not applicable/relevant for your study

"A Web-based Virtual Environment Behavioral Intervention"

1a-ii) Non-web-based components or important co-interventions in title

Mention non-web-based components or important co-interventions in title, if any (e.g., “with telephone support”).

	1	2	3	4	5	
subitem not at all important	(•)	( )	( )	( )	( )	essential

Clear selection

Does your paper address subitem 1a-ii?

Copy and paste relevant sections from manuscript title (include quotes in quotation marks "like this" to indicate direct quotes from your manuscript), or elaborate on this item by providing additional information not in the ms, or briefly explain why the item is not applicable/relevant for your study

We did not include this it was not relevant.

Your response is too large. Try shortening some answers.

1a-iii) Primary condition or target group in the title

Mention primary condition or target group in the title, if any (e.g., “for children with Type I Diabetes”) Example: A Web-based and Mobile Intervention with Telephone Support for Children with Type I Diabetes: Randomized Controlled Trial

	1	2	3	4	5	
subitem not at all important	○	○	○	○	◉	essential

Clear selection

Does your paper address subitem 1a-iii? *

Copy and paste relevant sections from manuscript title (include quotes in quotation marks "like this" to indicate direct quotes from your manuscript), or elaborate on this item by providing additional information not in the ms, or briefly explain why the item is not applicable/relevant for your study

"CVD and Metabolic Disease Prevention Education"

1b) ABSTRACT: Structured summary of trial design, methods, results, and conclusions

NPT extension: Description of experimental treatment, comparator, care providers, centers, and blinding status.

Your response is too large. Try shortening some answers.

1b-i) Key features/functionalities/components of the intervention and comparator in the METHODS section of the ABSTRACT

Mention key features/functionalities/components of the intervention and comparator in the abstract. If possible, also mention theories and principles used for designing the site. Keep in mind the needs of systematic reviewers and indexers by including important synonyms. (Note: Only report in the abstract what the main paper is reporting. If this information is missing from the main body of text, consider adding it)

	1	2	3	4	5	
subitem not at all important	○	○	○	○	◉	essential

Clear selection

Does your paper address subitem 1b-i? *

Copy and paste relevant sections from the manuscript abstract (include quotes in quotation marks "like this" to indicate direct quotes from your manuscript), or elaborate on this item by providing additional information not in the ms, or briefly explain why the item is not applicable/relevant for your study

The LEARN intervention included an orientation packet detailing step-by-step login instructions for the VE (Figure 1), provided a description of the VE districts (e.g., lobby, food court, plaza, and reflection garden), and outlined the activities available in each area. Its primary purpose was to familiarize participants with the VE's features and to facilitate basic troubleshooting. We also provided a suite of digital support materials. A student research assistant produced a series of brief tutorial videos using PowerPoint (each under six minutes) covering essential troubleshooting, navigation strategies, and "tips and tricks" for effective engagement within the VE (Figure 2). These orientation videos were distributed via email and posted on the VEs internal message boards.

Your response is too large. Try shortening some answers.

1b-ii) Level of human involvement in the METHODS section of the ABSTRACT

Clarify the level of human involvement in the abstract, e.g., use phrases like “fully automated” vs. “therapist/nurse/care provider/physician-assisted” (mention number and expertise of providers involved, if any). (Note: Only report in the abstract what the main paper is reporting. If this information is missing from the main body of text, consider adding it)

	1	2	3	4	5	
subitem not at all important	○	○	○	○	○	essential

Does your paper address subitem 1b-ii?

Copy and paste relevant sections from the manuscript abstract (include quotes in quotation marks "like this" to indicate direct quotes from your manuscript), or elaborate on this item by providing additional information not in the ms, or briefly explain why the item is not applicable/relevant for your study

Your answer

Your response is too large. Try shortening some answers.

1b-iii) Open vs. closed, web-based (self-assessment) vs. face-to-face assessments in the METHODS section of the ABSTRACT

Mention how participants were recruited (online vs. offline), e.g., from an open access website or from a clinic or a closed online user group (closed usergroup trial), and clarify if this was a purely web-based trial, or there were face-to-face components (as part of the intervention or for assessment). Clearly say if outcomes were self-assessed through questionnaires (as common in web-based trials). Note: In traditional offline trials, an open trial (open-label trial) is a type of clinical trial in which both the researchers and participants know which treatment is being administered. To avoid confusion, use “blinded” or “unblinded” to indicated the level of blinding instead of “open”, as “open” in web-based trials usually refers to “open access” (i.e. participants can self-enrol). (Note: Only report in the abstract what the main paper is reporting. If this information is missing from the main body of text, consider adding it)

	1	2	3	4	5	
subitem not at all important	◉	○	○	○	○	essential

Clear selection

Does your paper address subitem 1b-iii?

Copy and paste relevant sections from the manuscript abstract (include quotes in quotation marks "like this" to indicate direct quotes from your manuscript), or elaborate on this item by providing additional information not in the ms, or briefly explain why the item is not applicable/relevant for your study

"Self-reported survey data collection occurred through scheduled phone calls with the study coordinator at baseline, 3-month, and 6-month time points. The coordinator entered participant responses into REDCap, which included demographic, behavioral, and health indicators. Survey data collection took between 60 and 90 minutes. Acceptability data were assessed using engagement metrics derived from the virtual environment platform's metadata. This metadata comprised participant logins and log file analysis, which are digital records capturing activities within the virtual environment. This information provided insights into login frequency and duration of platform use during the study period, logged under each participant's avatar pseudonym."

Your response is too large. Try shortening some answers.

1b-iv) RESULTS section in abstract must contain use data

Report number of participants enrolled/assessed in each group, the use/uptake of the intervention (e.g., attrition/adherence metrics, use over time, number of logins etc.), in addition to primary/secondary outcomes. (Note: Only report in the abstract what the main paper is reporting. If this information is missing from the main body of text, consider adding it)

	1	2	3	4	5	
subitem not at all important	○	○	○	○	◉	essential

Clear selection

Does your paper address subitem 1b-iv?

Copy and paste relevant sections from the manuscript abstract (include quotes in quotation marks "like this" to indicate direct quotes from your manuscript), or elaborate on this item by providing additional information not in the ms, or briefly explain why the item is not applicable/relevant for your study

"Figure 5 CONSORT diagram provides summary of feasibility results. A total of 170 individuals were screened and assessed for eligibility, of which 92 were excluded (n=46 did not meet inclusion criteria, and n=46 declined to participate). In the RCT, 78 of the targeted 80 participants were enrolled (97.5%), demonstrating successful recruitment. Of these 78 participants, 40 entered the intervention group and 38 entered the wait-list control group.

Acceptability:
Table 2 provides a summary of acceptability results. Participants logged a total of 110 sessions, averaging 1 hour and 50 minutes each. Quests were accessed 14 times with the most frequently visited quests located in the fitness district (64.0%), followed by the grocery district (14.0%), and the pharmacy, bookstore, and outlet districts (7.0% each). The most engaged content were nutrition (146 engagements), oral health (138 engagements), fitness tips and videos (82 engagements), and relaxation techniques (75 engagements)."

Your response is too large. Try shortening some answers.

1b-v) CONCLUSIONS/DISCUSSION in abstract for negative trials

Conclusions/Discussions in abstract for negative trials: Discuss the primary outcome - if the trial is negative (primary outcome not changed), and the intervention was not used, discuss whether negative results are attributable to lack of uptake and discuss reasons. (Note: Only report in the abstract what the main paper is reporting. If this information is missing from the main body of text, consider adding it)

	1	2	3	4	5	
subitem not at all important	○	○	○	○	◉	essential

Clear selection

Does your paper address subitem 1b-v?

Copy and paste relevant sections from the manuscript abstract (include quotes in quotation marks "like this" to indicate direct quotes from your manuscript), or elaborate on this item by providing additional information not in the ms, or briefly explain why the item is not applicable/relevant for your study

"The LEARN Study represents the first known effort to assess the feasibility and acceptability of a virtual environment for cardiovascular and metabolic disease prevention education among men living with HIV.

The findings of the LEARN RCT demonstrated feasibility, as we enrolled 78 participants, achieving 98% of the targeted sample size. This outcome is noteworthy given that approximately 25% of randomized controlled trials fail to meet recruitment goals, even with extended trial periods.53 The high enrollment rate reflects the effectiveness of multiple recruitment strategies employed, including community partnerships, digital outreach through MyChart messaging campaigns, research listservs, and targeted communications. Retention presented challenges typically associated with tech-driven longitudinal research, with a final retention rate of 61.5% at the 6-month assessment. While modest, our retention outcome exceeded the typical rates observed in remote digital health initiatives, which peak around 56%.54-56 Attrition primarily occurred prior to baseline data collection, with 19 participants (24%) withdrawing after verbal consent, consistent with research indicating that more than half of discontinuations in digital health studies occur within the first week.57

Your response is too large. Try shortening some answers.

2a) In INTRODUCTION: Scientific background and explanation of rationale

2a-i) Problem and the type of system/solution

Describe the problem and the type of system/solution that is object of the study: intended as stand-alone intervention vs. incorporated in broader health care program? Intended for a particular patient population? Goals of the intervention, e.g., being more cost-effective to other interventions, replace or complement other solutions? (Note: Details about the intervention are provided in “Methods” under 5)

	1	2	3	4	5	
subitem not at all important	○	○	○	○	◉	essential

Clear selection

Your response is too large. Try shortening some answers.

Does your paper address subitem 2a-i? *

Copy and paste relevant sections from the manuscript (include quotes in quotation marks "like this" to indicate direct quotes from your manuscript), or elaborate on this item by providing additional information not in the ms, or briefly explain why the item is not applicable/relevant for your study

"Innovative approaches to lowering risk of CVD among men who are living with HIV, including behavioral prevention, education, and technological strategies, are essential to address pressing health issues in this population. In 2024, a study by the Pew Research Center indicated that the digital divide has significantly narrowed, with up to 90% of adults ages 65 and older and 91% of those living in poverty reported using the internet.20 A 2022 representative national survey by AARP found that 45% of individuals aged 50 and older engage in video gaming to maintain mental sharpness, reduce stress, entertain themselves, and solve problems.21 Research using serious games has been used for social connection and education post stroke,22 diabetes self-management23 and VR-based exercise in persons with diabetes,24 self-management and lifestyle changes in those with bipolar disorder,25 and cardiac rehabilitation,26. These findings highlight the potential for technology-driven behavioral interventions to serve as effective tools for health education and engagement, particularly in populations at risk, such as men living with HIV.

The Second Life Impacts Diabetes Education & Self-Management (SLIDES)27 study and the Learning in a Virtual Environment (LIVE©)28 platform were disease-agnostic virtual environments (VE) previously tested for diabetes self-management. Our team modified the LIVE© platform and shifted the focus and content to CVD and metabolic disease prevention education for sexual minority men living with HIV, entitled, “The LEARN study” (“LEveraging A ViRtual EnviroNment to Enhance Prevention of HIV-related Comorbidities”). Briefly, the LEARN study incorporated gamification and educational quests to enhance participant engagement while increasing awareness of cardiovascular health, risk factors, and effective prevention strategies. The platform was personalized for cultural salience and content through formative work described elsewhere29. The LEARN aims were: (a) to evaluate the feasibility and acceptability of this pilot intervention in persons living with HIV and to (b) examine preliminary efficacy of the intervention across health indicators.

Your response is too large. Try shortening some answers.

2a-ii) Scientific background, rationale: What is known about the (type of) system

Scientific background, rationale: What is known about the (type of) system that is the object of the study (be sure to discuss the use of similar systems for other conditions/diagnoses, if appropiate), motivation for the study, i.e. what are the reasons for and what is the context for this specific study, from which stakeholder viewpoint is the study performed, potential impact of findings [2]. Briefly justify the choice of the comparator.

	1	2	3	4	5	
subitem not at all important	○	○	○	○	◉	essential

Clear selection

Does your paper address subitem 2a-ii? *

Copy and paste relevant sections from the manuscript (include quotes in quotation marks "like this" to indicate direct quotes from your manuscript), or elaborate on this item by providing additional information not in the ms, or briefly explain why the item is not applicable/relevant for your study

"The Second Life Impacts Diabetes Education & Self-Management (SLIDES)27 study and the Learning in a Virtual Environment (LIVE©)28 platform were disease-agnostic virtual environments (VE) previously tested for diabetes self-management. Our team modified the LIVE© platform and shifted the focus and content to CVD and metabolic disease prevention education for sexual minority men living with HIV, entitled, “The LEARN study” (“LEveraging A ViRtual EnviroNment to Enhance Prevention of HIV-related Comorbidities”). Briefly, the LEARN study incorporated gamification and educational quests to enhance participant engagement while increasing awareness of cardiovascular health, risk factors, and effective prevention strategies. The platform was personalized for cultural salience and content through formative work described elsewhere29. The LEARN aims were: (a) to evaluate the feasibility and acceptability of this pilot intervention in persons living with HIV and to (b) examine preliminary efficacy of the intervention across health indicators."

2b) In INTRODUCTION: Specific objectives or hypotheses

Your response is too large. Try shortening some answers.

Does your paper address CONSORT subitem 2b? *

Copy and paste relevant sections from the manuscript (include quotes in quotation marks "like this" to indicate direct quotes from your manuscript), or elaborate on this item by providing additional information not in the ms, or briefly explain why the item is not applicable/relevant for your study

"The LEARN aims were: (a) to evaluate the feasibility and acceptability of this pilot intervention in persons living with HIV and to (b) examine preliminary efficacy of the intervention across health indicators."

METHODS

3a) Description of trial design (such as parallel, factorial) including allocation ratio

Your response is too large. Try shortening some answers.

Does your paper address CONSORT subitem 3a? *

Copy and paste relevant sections from the manuscript (include quotes in quotation marks "like this" to indicate direct quotes from your manuscript), or elaborate on this item by providing additional information not in the ms, or briefly explain why the item is not applicable/relevant for your study

"To the team's best knowledge, the LEARN Study was the first wait-list control randomized trial to test the feasibility and acceptability of a virtual environment for CVD and metabolic-disease prevention education in men with HIV.30,31 The waitlist design was advantageous to mitigate ethical dilemmas for the control group, as all participants would receive the intervention, and for pilot and efficacy trials, where no standard care treatment was available.32 Prior to the pilot intervention, our team conducted online-only qualitative interviews with n=15 individuals and beta testing of the VE with n=10 individuals. Findings about VE design and personalization leading to the LEARN clinical trial can be found elsewhere.30,31,33,34 Data suggested that participants were enthusiastic about the potential of a VE to address health risks associated with HIV and wanted more representation in avatar customizations. In this current study, enrolled individuals participated in the pilot trial over the course of 6-months based on their random assignment, and with outcomes measured at 3 timepoints: baseline, 3-months, and 6-months." "Between July 2023 and December 2024, individuals were screened to participate. Individuals who met eligibility criteria were provided informed consent to the research coordinator over the phone. Once consented, participants were allocated to immediate access in the virtual environment or to a wait-list control arm using permuted-block randomization with varying block sizes to ensure allocation concealment and balanced allocation to the intervention and control groups over the study period.37 Additionally, the coordinator verified each participant's identity through confirmatory phone calls and email confirmations to prevent individuals from enrolling multiple times."

3b) Important changes to methods after trial commencement (such as eligibility criteria), with reasons

Does your paper address CONSORT subitem 3b? *

Copy and paste relevant sections from the manuscript (include quotes in quotation marks "like this" to indicate direct quotes from your manuscript), or elaborate on this item by providing additional information not in the ms, or briefly explain why the item is not applicable/relevant for your study

we have none to report that we had to change

Your response is too large. Try shortening some answers.

3b-i) Bug fixes, Downtimes, Content Changes

Bug fixes, Downtimes, Content Changes: ehealth systems are often dynamic systems. A description of changes to methods therefore also includes important changes made on the intervention or comparator during the trial (e.g., major bug fixes or changes in the functionality or content) (5-iii) and other “unexpected events” that may have influenced study design such as staff changes, system failures/downtimes, etc. [2].

	1	2	3	4	5	
subitem not at all important	(•)	( )	( )	( )	( )	essential

Clear selection

Does your paper address subitem 3b-i?

Copy and paste relevant sections from the manuscript (include quotes in quotation marks "like this" to indicate direct quotes from your manuscript), or elaborate on this item by providing additional information not in the ms, or briefly explain why the item is not applicable/relevant for your study

we have none to report that we had to change

4a) Eligibility criteria for participants

Does your paper address CONSORT subitem 4a? *

Copy and paste relevant sections from the manuscript (include quotes in quotation marks "like this" to indicate direct quotes from your manuscript), or elaborate on this item by providing additional information not in the ms, or briefly explain why the item is not applicable/relevant for your study

"Eligibility criteria were: 1) ages 30 and up, 2) male sex; 3) HIV serostatus positive; 4) ability to participate; 5) have no medical history of serious complications, such as heart attack, stroke, or cognitive impairment. "

Your response is too large. Try shortening some answers.

4a-i) Computer / Internet literacy

Computer / Internet literacy is often an implicit “de facto” eligibility criterion - this should be explicitly clarified.

	1	2	3	4	5	
subitem not at all important	○	○	○	○	○	essential

Does your paper address subitem 4a-i?

Copy and paste relevant sections from the manuscript (include quotes in quotation marks "like this" to indicate direct quotes from your manuscript), or elaborate on this item by providing additional information not in the ms, or briefly explain why the item is not applicable/relevant for your study

Your answer

4a-ii) Open vs. closed, web-based vs. face-to-face assessments:

Open vs. closed, web-based vs. face-to-face assessments: Mention how participants were recruited (online vs. offline), e.g., from an open access website or from a clinic, and clarify if this was a purely web-based trial, or there were face-to-face components (as part of the intervention or for assessment), i.e., to what degree got the study team to know the participant. In online-only trials, clarify if participants were quasi-anonymous and whether having multiple identities was possible or whether technical or logistical measures (e.g., cookies, email confirmation, phone calls) were used to detect/prevent these.

	1	2	3	4	5	
subitem not at all important	○	○	○	○	◉	essential

Clear selection

Your response is too large. Try shortening some answers.

Does your paper address subitem 4a-ii? *

Copy and paste relevant sections from the manuscript (include quotes in quotation marks "like this" to indicate direct quotes from your manuscript), or elaborate on this item by providing additional information not in the ms, or briefly explain why the item is not applicable/relevant for your study

"We collaborated with the Yale Center for Clinical Investigation to support our clinical trial. A research coordinator with expertise in clinical trials screening, enrollment/consent, data collection, tracking, documentation, and regulatory compliance supported the team throughout the study.

To enhance retention, the study coordinator maintained regular contact with participants by sending voicemail and email reminders regarding upcoming data collection time points. "Self-reported survey data collection occurred through scheduled phone calls with the study coordinator at baseline, 3-month, and 6-month time points. The coordinator entered participant responses into REDCap, which included demographic, behavioral, and health indicators. Survey data collection took between 60 and 90 minutes. Acceptability data were assessed using engagement metrics derived from the virtual environment platform's metadata. This metadata comprised participant logins and log file analysis, which are digital records capturing activities within the virtual environment. This information provided insights into login frequency and duration of platform use during the study period, logged under each participant's avatar pseudonym."

4a-iii) Information giving during recruitment

Information given during recruitment. Specify how participants were briefed for recruitment and in the informed consent procedures (e.g., publish the informed consent documentation as appendix, see also item X26), as this information may have an effect on user self-selection, user expectation and may also bias results.

	1	2	3	4	5	
subitem not at all important	○	○	○	○	◉	essential

Clear selection

Your response is too large. Try shortening some answers.

Does your paper address subitem 4a-iii?

Copy and paste relevant sections from the manuscript (include quotes in quotation marks "like this" to indicate direct quotes from your manuscript), or elaborate on this item by providing additional information not in the ms, or briefly explain why the item is not applicable/relevant for your study

"We collaborated with the Yale Center for Clinical Investigation to support our clinical trial. A research coordinator with expertise in clinical trials screening, enrollment/consent, data collection, tracking, documentation, and regulatory compliance supported the team throughout the study." "Individuals who met eligibility criteria were provided informed consent to the research coordinator over the phone. "

4b) Settings and locations where the data were collected

Does your paper address CONSORT subitem 4b? *

Copy and paste relevant sections from the manuscript (include quotes in quotation marks "like this" to indicate direct quotes from your manuscript), or elaborate on this item by providing additional information not in the ms, or briefly explain why the item is not applicable/relevant for your study

"Self-reported survey data collection occurred through scheduled phone calls with the study coordinator at baseline, 3-month, and 6-month time points. The coordinator entered participant responses into REDCap, which included demographic, behavioral, and health indicators. Survey data collection took between 60 and 90 minutes. Acceptability data were assessed using engagement metrics derived from the virtual environment platform's metadata. This metadata comprised participant logins and log file analysis, which are digital records capturing activities within the virtual environment. This information provided insights into login frequency and duration of platform use during the study period, logged under each participant's avatar pseudonym"

Your response is too large. Try shortening some answers.

4b-i) Report if outcomes were (self-)assessed through online questionnaires

Clearly report if outcomes were (self-)assessed through online questionnaires (as common in web-based trials) or otherwise.

	1	2	3	4	5	
subitem not at all important	○	○	○	○	◉	essential

Clear selection

Does your paper address subitem 4b-i? *

Copy and paste relevant sections from the manuscript (include quotes in quotation marks "like this" to indicate direct quotes from your manuscript), or elaborate on this item by providing additional information not in the ms, or briefly explain why the item is not applicable/relevant for your study

"Self-reported survey data collection occurred through scheduled phone calls with the study coordinator at baseline, 3-month, and 6-month time points. The coordinator entered participant responses into REDCap, which included demographic, behavioral, and health indicators. Survey data collection took between 60 and 90 minutes."

4b-ii) Report how institutional affiliations are displayed

Report how institutional affiliations are displayed to potential participants [on ehealth media], as affiliations with prestigious hospitals or universities may affect volunteer rates, use, and reactions with regards to an intervention.(Not a required item – describe only if this may bias results)

	1	2	3	4	5	
subitem not at all important	◉	○	○	○	○	essential

Clear selection

Your response is too large. Try shortening some answers.

Does your paper address subitem 4b-ii?

Copy and paste relevant sections from the manuscript (include quotes in quotation marks "like this" to indicate direct quotes from your manuscript), or elaborate on this item by providing additional information not in the ms, or briefly explain why the item is not applicable/relevant for your study

Your answer

5) The interventions for each group with sufficient details to allow replication, including how and when they were actually administered

5-i) Mention names, credential, affiliations of the developers, sponsors, and owners

Mention names, credential, affiliations of the developers, sponsors, and owners [6] (if authors/evaluators are owners or developer of the software, this needs to be declared in a "Conflict of interest" section or mentioned elsewhere in the manuscript).

	1	2	3	4	5	
subitem not at all important	○	○	○	○	◉	essential

Clear selection

Does your paper address subitem 5-i?

Copy and paste relevant sections from the manuscript (include quotes in quotation marks "like this" to indicate direct quotes from your manuscript), or elaborate on this item by providing additional information not in the ms, or briefly explain why the item is not applicable/relevant for your study

"Authors CJ and AV are the investigators who led the development of the Learning in a Virtual Environment (LIVE) platform. "

Your response is too large. Try shortening some answers.

5-ii) Describe the history/development process

Describe the history/development process of the application and previous formative evaluations (e.g., focus groups, usability testing), as these will have an impact on adoption/use rates and help with interpreting results.

	1	2	3	4	5	
subitem not at all important	○	○	○	○	◉	essential

Clear selection

Does your paper address subitem 5-ii?

Copy and paste relevant sections from the manuscript (include quotes in quotation marks "like this" to indicate direct quotes from your manuscript), or elaborate on this item by providing additional information not in the ms, or briefly explain why the item is not applicable/relevant for your study

"The Second Life Impacts Diabetes Education & Self-Management (SLIDES)27 study and the Learning in a Virtual Environment (LIVE©)28 platform were disease-agnostic virtual environments (VE) previously tested for diabetes self-management. Our team modified the LIVE© platform and shifted the focus and content to CVD and metabolic disease prevention education for sexual minority men living with HIV, entitled, “The LEARN study” (“LEveraging A ViRtual EnviroNment to Enhance Prevention of HIV-related Comorbidities”). Briefly, the LEARN study incorporated gamification and educational quests to enhance participant engagement while increasing awareness of cardiovascular health, risk factors, and effective prevention strategies. The platform was personalized for cultural salience and content through formative work described elsewhere29. The LEARN aims were: (a) to evaluate the feasibility and acceptability of this pilot intervention in persons living with HIV and to (b) examine preliminary efficacy of the intervention across health indicators. "

Your response is too large. Try shortening some answers.

5-iii) Revisions and updating

Revisions and updating. Clearly mention the date and/or version number of the application/intervention (and comparator, if applicable) evaluated, or describe whether the intervention underwent major changes during the evaluation process, or whether the development and/or content was “frozen” during the trial. Describe dynamic components such as news feeds or changing content which may have an impact on the replicability of the intervention (for unexpected events see item 3b).

	1	2	3	4	5	
subitem not at all important	◉	○	○	○	○	essential

Clear selection

Does your paper address subitem 5-iii?

Copy and paste relevant sections from the manuscript (include quotes in quotation marks "like this" to indicate direct quotes from your manuscript), or elaborate on this item by providing additional information not in the ms, or briefly explain why the item is not applicable/relevant for your study

Your answer

5-iv) Quality assurance methods

Provide information on quality assurance methods to ensure accuracy and quality of information provided [1], if applicable.

	1	2	3	4	5	
subitem not at all important	○	○	○	○	◉	essential

Clear selection

Your response is too large. Try shortening some answers.

Does your paper address subitem 5-iv?

Copy and paste relevant sections from the manuscript (include quotes in quotation marks "like this" to indicate direct quotes from your manuscript), or elaborate on this item by providing additional information not in the ms, or briefly explain why the item is not applicable/relevant for your study

"The methodological rigor of this study balances the limitations with notable strengths. For instance, intervention fidelity was maintained through a comprehensive approach addressing five components, 1) intervention design, 2) study staff training, 3) intervention delivery, 4) participant receipt, and 5) outcome assessment. First, all study staff completed ethical research training, engaged in role-playing for participant recruitment and enrollment, and followed a detailed scope of work document outlining research-related tasks and responsibilities. Second, participant receipt of the intervention was objectively measured using login frequency, engagement duration, and log file data capturing specific activities within the virtual environment. Third, the study coordinator, trained through the Clinical and Translational Science Award program, ensured consistent participant screening, enrollment, consent, and data collection using standardized scripts. Fourth, a statistician designed the permuted block randomization, and although the principal investigator had access to participant allocation, the PI and study coordinator did not engage in data analysis. This was done to minimize bias in outcome ascertainment.64 Fifth, data collection and storage utilized REDCap, a HIPAA-compliant, validated platform recognized for reliability in clinical research. Sixth, the study's educational content was grounded in the American Heart Association's Life's Essential 8 framework36 for cardiovascular health, lending credibility to the prevention messaging delivered through the virtual environment.57 Last, the novel approach to cardiovascular and metabolic disease prevention education utilizing gamification and interactive quests distinguishes this intervention from traditional clinical patient education. The high enrollment rate, methodological rigor, and use of validated measures enhance the validity of findings. The sample's demographic composition (i.e. Black and Hispanic men living with HIV) addresses a critical gap in clinical trial representation. Engagement metrics demonstrating nearly two-hour average sessions highlight participants' favorable reception of the virtual environment content, supporting the potential of this approach for reaching underserved populations with innovative health education."

Your response is too large. Try shortening some answers.

5-v) Ensure replicability by publishing the source code, and/or providing screenshots/screen-capture video, and/or providing flowcharts of the algorithms used

Ensure replicability by publishing the source code, and/or providing screenshots/screen-capture video, and/or providing flowcharts of the algorithms used. Replicability (i.e., other researchers should in principle be able to replicate the study) is a hallmark of scientific reporting.

	1	2	3	4	5	
subitem not at all important	○	○	○	○	●	essential

Clear selection

Does your paper address subitem 5-v?

Copy and paste relevant sections from the manuscript (include quotes in quotation marks "like this" to indicate direct quotes from your manuscript), or elaborate on this item by providing additional information not in the ms, or briefly explain why the item is not applicable/relevant for your study

"Figure 5 CONSORT diagram provides summary of results. A total of 170 individuals were screened and assessed for eligibility, of which 92 were excluded (n=46 did not meet inclusion criteria, and n=46 declined to participate). In the RCT, 78 of the targeted 80 participants were enrolled (97.5%), demonstrating successful recruitment. Of these 78 participants, 40 entered the intervention group and 38 entered the wait-list control group."

Your response is too large. Try shortening some answers.

5-vi) Digital preservation

Digital preservation: Provide the URL of the application, but as the intervention is likely to change or disappear over the course of the years; also make sure the intervention is archived (Internet Archive, webcitation.org, and/or publishing the source code or screenshots/videos alongside the article). As pages behind login screens cannot be archived, consider creating demo pages which are accessible without login.

	1	2	3	4	5	
subitem not at all important	◉	○	○	○	○	essential

Clear selection

Does your paper address subitem 5-vi?

Copy and paste relevant sections from the manuscript (include quotes in quotation marks "like this" to indicate direct quotes from your manuscript), or elaborate on this item by providing additional information not in the ms, or briefly explain why the item is not applicable/relevant for your study

We used an instance of the VE. The platform was shut down a year ago by developers. Screenshots of VE are included in our JMIR protocol and 1st results papers.

5-vii) Access

Access: Describe how participants accessed the application, in what setting/context, if they had to pay (or were paid) or not, whether they had to be a member of specific group. If known, describe how participants obtained "access to the platform and Internet" [1]. To ensure access for editors/reviewers/readers, consider to provide a "backdoor" login account or demo mode for reviewers/readers to explore the application (also important for archiving purposes, see vi).

	1	2	3	4	5	
subitem not at all important	○	○	○	◉	○	essential

Clear selection

Your response is too large. Try shortening some answers.

Does your paper address subitem 5-vii? *

Copy and paste relevant sections from the manuscript (include quotes in quotation marks "like this" to indicate direct quotes from your manuscript), or elaborate on this item by providing additional information not in the ms, or briefly explain why the item is not applicable/relevant for your study

Participants were provided access after screening and consent by the study coordinator. They accessed a link we provided and logged in with credentials assigned. They were compensated for completion of measures at the three timepoints 0,3 6.

5-viii) Mode of delivery, features/functionalities/components of the intervention and comparator, and the theoretical framework

Describe mode of delivery, features/functionalities/components of the intervention and comparator, and the theoretical framework [6] used to design them (instructional strategy [1], behaviour change techniques, persuasive features, etc., see e.g., [7, 8] for terminology). This includes an in-depth description of the content (including where it is coming from and who developed it) [1]," whether [and how] it is tailored to individual circumstances and allows users to track their progress and receive feedback" [6]. This also includes a description of communication delivery channels and – if computer-mediated communication is a component – whether communication was synchronous or asynchronous [6]. It also includes information on presentation strategies [1], including page design principles, average amount of text on pages, presence of hyperlinks to other resources, etc. [1].

	1	2	3	4	5	
subitem not at all important	○	○	○	○	◉	essential

Clear selection

Your response is too large. Try shortening some answers.

Does your paper address subitem 5-viii? *

Copy and paste relevant sections from the manuscript (include quotes in quotation marks "like this" to indicate direct quotes from your manuscript), or elaborate on this item by providing additional information not in the ms, or briefly explain why the item is not applicable/relevant for your study

"Participants were allocated to the virtual environment intervention (n = 40) or a wait-list control arm (n = 38). The LEARN intervention included an orientation packet detailing step-by-step login instructions for the VE (Figure 1), provided a description of the VE districts (e.g., lobby, food court, plaza, and reflection garden), and outlined the activities available in each area. Its primary purpose was to familiarize participants with the VE's features and to facilitate basic troubleshooting. We also provided a suite of digital support materials. A student research assistant produced a series of brief tutorial videos using PowerPoint (each under six minutes) covering essential troubleshooting, navigation strategies, and "tips and tricks" for effective engagement within the VE (Figure 2). These orientation videos were distributed via email and posted on the VEs internal message boards.
Once onboarded, participants engaged in interactive, self-directed exploration in the VE. They customized their avatar's (hair color, skin color, clothing, and footwear) to reflect personal preferences and identity considerations, navigated across multiple VE districts, and encountered targeted health content designed to promote behavioral modification. Within each district, participants were presented with information and images relevant to cardiovascular and metabolic health, such as making dietary choices in the food court or exploring physical activity resources in the plaza to reinforce key prevention messaging in an immersive context.
"

5-ix) Describe use parameters

Describe use parameters (e.g., intended "doses" and optimal timing for use). Clarify what instructions or recommendations were given to the user, e.g., regarding timing, frequency, heaviness of use, if any, or was the intervention used ad libitum.

	1	2	3	4	5	
subitem not at all important	○	○	○	○	○	essential

Your response is too large. Try shortening some answers.

Does your paper address subitem 5-ix?

Copy and paste relevant sections from the manuscript (include quotes in quotation marks "like this" to indicate direct quotes from your manuscript), or elaborate on this item by providing additional information not in the ms, or briefly explain why the item is not applicable/relevant for your study

Your answer

5-x) Clarify the level of human involvement

Clarify the level of human involvement (care providers or health professionals, also technical assistance) in the e-intervention or as co-intervention (detail number and expertise of professionals involved, if any, as well as “type of assistance offered, the timing and frequency of the support, how it is initiated, and the medium by which the assistance is delivered”. It may be necessary to distinguish between the level of human involvement required for the trial, and the level of human involvement required for a routine application outside of a RCT setting (discuss under item 21 – generalizability).

	1	2	3	4	5	
subitem not at all important	○	○	○	○	○	essential

Does your paper address subitem 5-x?

Copy and paste relevant sections from the manuscript (include quotes in quotation marks "like this" to indicate direct quotes from your manuscript), or elaborate on this item by providing additional information not in the ms, or briefly explain why the item is not applicable/relevant for your study

Your answer

Your response is too large. Try shortening some answers.

5-xi) Report any prompts/reminders used

Report any prompts/reminders used: Clarify if there were prompts (letters, emails, phone calls, SMS) to use the application, what triggered them, frequency etc. It may be necessary to distinguish between the level of prompts/reminders required for the trial, and the level of prompts/reminders for a routine application outside of a RCT setting (discuss under item 21 – generalizability).

	1	2	3	4	5	
subitem not at all important	○	○	○	○	◉	essential

Clear selection

Does your paper address subitem 5-xi? *

Copy and paste relevant sections from the manuscript (include quotes in quotation marks "like this" to indicate direct quotes from your manuscript), or elaborate on this item by providing additional information not in the ms, or briefly explain why the item is not applicable/relevant for your study

"To enhance retention, the study coordinator maintained regular contact with participants by sending voicemail and email reminders regarding upcoming data collection time points. Monthly newsletters were also distributed via email to foster general study communication (Figure 4)."

5-xii) Describe any co-interventions (incl. training/support)

Describe any co-interventions (incl. training/support): Clearly state any interventions that are provided in addition to the targeted eHealth intervention, as ehealth intervention may not be designed as stand-alone intervention. This includes training sessions and support [1]. It may be necessary to distinguish between the level of training required for the trial, and the level of training for a routine application outside of a RCT setting (discuss under item 21 – generalizability.

	1	2	3	4	5	
subitem not at all important	○	○	○	○	◉	essential

Your response is too large. Try shortening some answers.

Does your paper address subitem 5-xii? *

Copy and paste relevant sections from the manuscript (include quotes in quotation marks "like this" to indicate direct quotes from your manuscript), or elaborate on this item by providing additional information not in the ms, or briefly explain why the item is not applicable/relevant for your study

"First, all study staff completed ethical research training, engaged in role-playing for participant recruitment and enrollment, and followed a detailed scope of work document outlining research-related tasks and responsibilities. the study coordinator, trained through the Clinical and Translational Science Award program, ensured consistent participant screening, enrollment, consent, and data collection using standardized scripts."

6a) Completely defined pre-specified primary and secondary outcome measures, including how and when they were assessed

Your response is too large. Try shortening some answers.

Does your paper address CONSORT subitem 6a? *

Copy and paste relevant sections from the manuscript (include quotes in quotation marks "like this" to indicate direct quotes from your manuscript), or elaborate on this item by providing additional information not in the ms, or briefly explain why the item is not applicable/relevant for your study

"Measures:
Self-reported survey data collection occurred through scheduled phone calls with the study coordinator at baseline, 3-month, and 6-month time points. The coordinator entered participant responses into REDCap, which included demographic, behavioral, and health indicators.

Primary Outcomes:
The primary outcomes for this study were the feasibility and acceptability of the virtual environment. Feasibility was operationalized through recruitment and retention metrics. Recruitment metrics were assessed by tracking the proportion of individuals who enrolled from those initially contacted. Retention metrics were measured as the percentage of participants who completed the 3-month and 6-month follow-up assessments. Acceptability was evaluated through engagement metrics with the VE, quantified by calculating the total time participants engaged with the platform (in minutes). This included the duration spent visiting various districts (e.g., grocery, pharmacy, bookstore, and outlet districts) as well as health content modules (e.g., nutrition, oral health, fitness tips and videos, relaxation techniques).

Health Indicators:
Multiple cardiovascular health indicators were assessed at baseline, 3-month, and 6-month follow-ups using self-report measures aligned with the American Heart Association's Life's Essential Eight metrics for cardiovascular health.36 We assessed self-reported measures indicating history of hypertension status (yes/no) or diabetes (yes/no). Sleep was measured by hours of sleep per night, with adequacy evaluated against the recommended 7-9 hours.41 Body Mass Index (BMI) was calculated from self-reported height and weight (kg/m^2) and categorized as underweight (<18.5), normal weight (18.5-24.9), overweight (25.0-29.9), or overweight/obese (≥30.0).42 Nutritional intake was assessed through average daily servings of vegetables, fruit, and whole grains, along with weekly consumption of sweets, with adherence evaluated against recommendations of 3 servings of vegetables and 2.5 servings of fruit daily.43,44 Physical activity was assessed using the International Physical Activity Questionnaire (IPAQ), which measures self-reported data in terms of time spent in minutes per week, minutes doing walking, doing moderate activity, and doing vigorous activity.45 Mental health was evaluated via Patient Health Questionnaire-9 (PHQ-9) through measures of depressive symptoms, with participants categorized as having mild, moderate, or severe depression.46 Tobacco and e-cigarette use were assessed, including smoking status and quit attempts using the Behavioral Risk Factor Surveillance System Questionnaire.47

Your response is too large. Try shortening some answers.

6a-i) Online questionnaires: describe if they were validated for online use and apply CHERRIES items to describe how the questionnaires were designed/deployed

If outcomes were obtained through online questionnaires, describe if they were validated for online use and apply CHERRIES items to describe how the questionnaires were designed/deployed [9].

	1	2	3	4	5	
subitem not at all important	○	○	○	◉	○	essential

Clear selection

Does your paper address subitem 6a-i?

Copy and paste relevant sections from manuscript text

"Multiple cardiovascular health indicators were assessed at baseline, 3-month, and 6-month follow-ups using self-report measures aligned with the American Heart Association's Life's Essential Eight metrics for cardiovascular health.36 We assessed self-reported measures indicating history of hypertension status (yes/no) or diabetes (yes/no). Sleep was measured by hours of sleep per night, with adequacy evaluated against the recommended 7-9 hours.41 Body Mass Index (BMI) was calculated from self-reported height and weight (kg/m²) and categorized as underweight (<18.5), normal weight (18.5-24.9), overweight (25.0-29.9), or overweight/obese (≥30.0).42 Nutritional intake was assessed through average daily servings of vegetables, fruit, and whole grains, along with weekly consumption of sweets, with adherence evaluated against recommendations of 3 servings of vegetables and 2.5 servings of fruit daily.43,44 Physical activity was assessed using the International Physical Activity Questionnaire (IPAQ), which measures self-reported data in terms of time spent in minutes per week, minutes doing walking, doing moderate activity, and doing vigorous activity.45 Mental health was evaluated via Patient Health Questionnaire-9 (PHQ-9) through measures of depressive symptoms, with participants categorized as having mild, moderate, or severe depression.46 Tobacco and e-cigarette use were assessed, including smoking status and quit attempts using the Behavioral Risk Factor Surveillance System Questionnaire.47

Your response is too large. Try shortening some answers.

6a-ii) Describe whether and how “use” (including intensity of use/dosage) was defined/measured/monitored

Describe whether and how “use” (including intensity of use/dosage) was defined/measured/monitored (logins, logfile analysis, etc.). Use/adoption metrics are important process outcomes that should be reported in any ehealth trial.

	1	2	3	4	5	
subitem not at all important	○	○	○	○	○	essential

Does your paper address subitem 6a-ii?

Copy and paste relevant sections from manuscript text

Your answer

6a-iii) Describe whether, how, and when qualitative feedback from participants was obtained

Describe whether, how, and when qualitative feedback from participants was obtained (e.g., through emails, feedback forms, interviews, focus groups).

	1	2	3	4	5	
subitem not at all important	○	○	○	○	○	essential

Does your paper address subitem 6a-iii?

Copy and paste relevant sections from manuscript text

Your answer

Your response is too large. Try shortening some answers.

Does your paper address CONSORT subitem 6b? *

Copy and paste relevant sections from the manuscript (include quotes in quotation marks "like this" to indicate direct quotes from your manuscript), or elaborate on this item by providing additional information not in the ms, or briefly explain why the item is not applicable/relevant for your study

No changes to trial outcomes after commenced.

7a) How sample size was determined

NPT: When applicable, details of whether and how the clustering by care provides or centers was addressed

7a-i) Describe whether and how expected attrition was taken into account when calculating the sample size

Describe whether and how expected attrition was taken into account when calculating the sample size.

	1	2	3	4	5	
subitem not at all important	○	○	○	○	○	essential

Does your paper address subitem 7a-i?

Copy and paste relevant sections from manuscript title (include quotes in quotation marks "like this" to indicate direct quotes from your manuscript), or elaborate on this item by providing additional information not in the ms, or briefly explain why the item is not applicable/relevant for your study

Your answer

7b) When applicable, explanation of any interim analyses and stopping guidelines

Your response is too large. Try shortening some answers.

Does your paper address CONSORT subitem 7b? *

Copy and paste relevant sections from the manuscript (include quotes in quotation marks "like this" to indicate direct quotes from your manuscript), or elaborate on this item by providing additional information not in the ms, or briefly explain why the item is not applicable/relevant for your study

We did not have any interim analyses. This was a pilot study.

8a) Method used to generate the random allocation sequence

NPT: When applicable, how care providers were allocated to each trial group

Does your paper address CONSORT subitem 8a? *

Copy and paste relevant sections from the manuscript (include quotes in quotation marks "like this" to indicate direct quotes from your manuscript), or elaborate on this item by providing additional information not in the ms, or briefly explain why the item is not applicable/relevant for your study

Once consented, participants were allocated to immediate access in the virtual environment or to a wait-list control arm using permuted-block randomization with varying block sizes to ensure allocation concealment and balanced allocation to the intervention and control groups over the study period.37

8b) Type of randomisation; details of any restriction (such as blocking and block size)

Your response is too large. Try shortening some answers.

Does your paper address CONSORT subitem 8b? *

Copy and paste relevant sections from the manuscript (include quotes in quotation marks "like this" to indicate direct quotes from your manuscript), or elaborate on this item by providing additional information not in the ms, or briefly explain why the item is not applicable/relevant for your study

Once consented, participants were allocated to immediate access in the virtual environment or to a wait-list control arm using permuted-block randomization with varying block sizes to ensure allocation concealment and balanced allocation to the intervention and control groups over the study period.37

9) Mechanism used to implement the random allocation sequence (such as sequentially numbered containers), describing any steps taken to conceal the sequence until interventions were assigned

Does your paper address CONSORT subitem 9? *

Copy and paste relevant sections from the manuscript (include quotes in quotation marks "like this" to indicate direct quotes from your manuscript), or elaborate on this item by providing additional information not in the ms, or briefly explain why the item is not applicable/relevant for your study

Once consented, participants were allocated to immediate access in the virtual environment or to a wait-list control arm using permuted-block randomization with varying block sizes to ensure allocation concealment and balanced allocation to the intervention and control groups over the study period.37

10) Who generated the random allocation sequence, who enrolled participants, and who assigned participants to interventions

Your response is too large. Try shortening some answers.

Does your paper address CONSORT subitem 10? *

Copy and paste relevant sections from the manuscript (include quotes in quotation marks "like this" to indicate direct quotes from your manuscript), or elaborate on this item by providing additional information not in the ms, or briefly explain why the item is not applicable/relevant for your study

The statistician generated the random allocation sequence. The study coordinator enrolled participants. The randomization sequence allocated the participants to the immediate intervention or the wait-list.

11a) If done, who was blinded after assignment to interventions (for example, participants, care providers, those assessing outcomes) and how

NPT: Whether or not administering co-interventions were blinded to group assignment

11a-i) Specify who was blinded, and who wasn't

Specify who was blinded, and who wasn't. Usually, in web-based trials it is not possible to blind the participants [1, 3] (this should be clearly acknowledged), but it may be possible to blind outcome assessors, those doing data analysis or those administering co-interventions (if any).

	1	2	3	4	5	
subitem not at all important	○	○	○	○	◉	essential

Clear selection

Does your paper address subitem 11a-i? *

Copy and paste relevant sections from the manuscript (include quotes in quotation marks "like this" to indicate direct quotes from your manuscript), or elaborate on this item by providing additional information not in the ms, or briefly explain why the item is not applicable/relevant for your study

"a statistician designed the permuted block randomization, and although the principal investigator had access to participant allocation, the PI and study coordinator did not

Your response is too large. Try shortening some answers.

11a-ii) Discuss e.g., whether participants knew which intervention was the "intervention of interest" and which one was the "comparator"

Informed consent procedures (4a-ii) can create biases and certain expectations - discuss e.g., whether participants knew which intervention was the "intervention of interest" and which one was the "comparator".

	1	2	3	4	5	
subitem not at all important	○	○	○	○	○	essential

Does your paper address subitem 11a-ii?

Copy and paste relevant sections from the manuscript (include quotes in quotation marks "like this" to indicate direct quotes from your manuscript), or elaborate on this item by providing additional information not in the ms, or briefly explain why the item is not applicable/relevant for your study

Your answer

11b) If relevant, description of the similarity of interventions

(this item is usually not relevant for ehealth trials as it refers to similarity of a placebo or sham intervention to a active medication/intervention)

Does your paper address CONSORT subitem 11b? *

Copy and paste relevant sections from the manuscript (include quotes in quotation marks "like this" to indicate direct quotes from your manuscript), or elaborate on this item by providing additional information not in the ms, or briefly explain why the item is not applicable/relevant for your study

We did not have a placebo or sham.

Your response is too large. Try shortening some answers.

12a) Statistical methods used to compare groups for primary and secondary outcomes

NPT: When applicable, details of whether and how the clustering by care providers or centers was addressed

Does your paper address CONSORT subitem 12a? *

Copy and paste relevant sections from the manuscript (include quotes in quotation marks "like this" to indicate direct quotes from your manuscript), or elaborate on this item by providing additional information not in the ms, or briefly explain why the item is not applicable/relevant for your study

"Means and standard deviations (M, SDs) were used to describe continuous variables, while frequency and percentages (n, %) were used to describe categorical variables, as appropriate. We tested associations between outcome and demographic variables by group at baseline, using the Student's T-Test for continuous outcome variables, and a Chi-squared test of independence for categorical variables. For preliminary efficacy testing, health indicators were collected and analyzed longitudinally across three timepoints (baseline, 3-month, and 6-month) and compared intervention vs. wait-list control groups. Linear mixed-effects models were utilized with restricted maximum likelihood estimation to examine differences in each health indicator between intervention and control groups over time. The model included fixed effects for randomization group, visit time, and a group × time interaction, as well as age, years living with HIV, income category, and education category as covariates. A random intercept was included to account for within-subject correlation across repeated measurements. Type 3 tests of fixed effects were used to evaluate the significance of each term in the model. Differences of least squares means between intervention and control groups were estimated at each timepoint and are presented as effect size estimates with 95% confidence intervals. Statistical significance was set at α = 0.05. All analyses were conducted using SAS Version 9.4 (SAS Institute Inc, Cary, North Carolina)"

Your response is too large. Try shortening some answers.

12a-i) Imputation techniques to deal with attrition / missing values

Imputation techniques to deal with attrition / missing values: Not all participants will use the intervention/comparator as intended and attrition is typically high in ehealth trials. Specify how participants who did not use the application or dropped out from the trial were treated in the statistical analysis (a complete case analysis is strongly discouraged, and simple imputation techniques such as LOCF may also be problematic [4]).

	1	2	3	4	5	
subitem not at all important	○	○	○	○	◉	essential

Clear selection

Does your paper address subitem 12a-i? *

Copy and paste relevant sections from the manuscript (include quotes in quotation marks "like this" to indicate direct quotes from your manuscript), or elaborate on this item by providing additional information not in the ms, or briefly explain why the item is not applicable/relevant for your study

"Means and standard deviations (M, SDs) were used to describe continuous variables, while frequency and percentages (n, %) were used to describe categorical variables, as appropriate. We tested associations between outcome and demographic variables by group at baseline, using the Student's T-Test for continuous outcome variables, and a Chi-squared test of independence for categorical variables. For preliminary efficacy testing, health indicators were collected and analyzed longitudinally across three timepoints (baseline, 3-month, and 6-month) and compared intervention vs. wait-list control groups. Linear mixed-effects models were utilized with restricted maximum likelihood estimation to examine differences in each health indicator between intervention and control groups over time. The model included fixed effects for randomization group, visit time, and a group × time interaction, as well as age, years living with HIV, income category, and education category as covariates. A random intercept was included to account for within-subject correlation across repeated measurements. Type 3 tests of fixed effects were used to evaluate the significance of each term in the model. Differences of least squares means between intervention and control groups were estimated at each timepoint and are presented as effect size estimates with 95% confidence intervals. Statistical significance was set at α = 0.05. All analyses were conducted using SAS Version 9.4 (SAS Institute Inc, Cary, North Carolina)"

Your response is too large. Try shortening some answers.

12b) Methods for additional analyses, such as subgroup analyses and adjusted analyses

Does your paper address CONSORT subitem 12b? *

Copy and paste relevant sections from the manuscript (include quotes in quotation marks "like this" to indicate direct quotes from your manuscript), or elaborate on this item by providing additional information not in the ms, or briefly explain why the item is not applicable/relevant for your study

"Means and standard deviations (M, SDs) were used to describe continuous variables, while frequency and percentages (n, %) were used to describe categorical variables, as appropriate. We tested associations between outcome and demographic variables by group at baseline, using the Student's T-Test for continuous outcome variables, and a Chi-squared test of independence for categorical variables. For preliminary efficacy testing, health indicators were collected and analyzed longitudinally across three timepoints (baseline, 3-month, and 6-month) and compared intervention vs. wait-list control groups. Linear mixed-effects models were utilized with restricted maximum likelihood estimation to examine differences in each health indicator between intervention and control groups over time. The model included fixed effects for randomization group, visit time, and a group × time interaction, as well as age, years living with HIV, income category, and education category as covariates. A random intercept was included to account for within-subject correlation across repeated measurements. Type 3 tests of fixed effects were used to evaluate the significance of each term in the model. Differences of least squares means between intervention and control groups were estimated at each timepoint and are presented as effect size estimates with 95% confidence intervals. Statistical significance was set at $\alpha = 0.05$. All analyses were conducted using SAS Version 9.4 (SAS Institute Inc, Cary, North Carolina)"

X26) REB/IRB Approval and Ethical Considerations [recommended as subheading under "Methods"] (not a CONSORT item)

Your response is too large. Try shortening some answers.

X26-i) Comment on ethics committee approval

	1	2	3	4	5	
subitem not at all important	○	○	○	○	◉	essential

Clear selection

Does your paper address subitem X26-i?

Copy and paste relevant sections from the manuscript (include quotes in quotation marks "like this" to indicate direct quotes from your manuscript), or elaborate on this item by providing additional information not in the ms, or briefly explain why the item is not applicable/relevant for your study

"All procedures were approved by the Yale Human Research Protection Program (IRB#: 2000031403)."

x26-ii) Outline informed consent procedures

Outline informed consent procedures e.g., if consent was obtained offline or online (how? Checkbox, etc.?), and what information was provided (see 4a-ii). See [6] for some items to be included in informed consent documents.

	1	2	3	4	5	
subitem not at all important	○	○	○	○	○	essential

Does your paper address subitem X26-ii?

Copy and paste relevant sections from the manuscript (include quotes in quotation marks "like this" to indicate direct quotes from your manuscript), or elaborate on this item by providing additional information not in the ms, or briefly explain why the item is not applicable/relevant for your study

Your response is too large. Try shortening some answers.

X26-iii) Safety and security procedures

Safety and security procedures, incl. privacy considerations, and any steps taken to reduce the likelihood or detection of harm (e.g., education and training, availability of a hotline)

	1	2	3	4	5	
subitem not at all important	○	○	○	○	○	essential

Does your paper address subitem X26-iii?

Copy and paste relevant sections from the manuscript (include quotes in quotation marks "like this" to indicate direct quotes from your manuscript), or elaborate on this item by providing additional information not in the ms, or briefly explain why the item is not applicable/relevant for your study

Your answer

RESULTS

13a) For each group, the numbers of participants who were randomly assigned, received intended treatment, and were analysed for the primary outcome

NPT: The number of care providers or centers performing the intervention in each group and the number of patients treated by each care provider in each center

Does your paper address CONSORT subitem 13a? *

Copy and paste relevant sections from the manuscript (include quotes in quotation marks "like this" to indicate direct quotes from your manuscript), or elaborate on this item by providing additional information not in the ms, or briefly explain why the item is not applicable/relevant for your study

Please see our CONSORT diagram.

Your response is too large. Try shortening some answers.

13b) For each group, losses and exclusions after randomisation, together with reasons

Does your paper address CONSORT subitem 13b? (NOTE: Preferably, this is shown in a CONSORT flow diagram) *

Copy and paste relevant sections from the manuscript (include quotes in quotation marks "like this" to indicate direct quotes from your manuscript), or elaborate on this item by providing additional information not in the ms, or briefly explain why the item is not applicable/relevant for your study

"The final analytic sample size for this study accounted for attrition. A total of 59 participants (n=31 in the intervention and n=28 in the wait-list control group) were included in the final analytic sample, following attrition primarily occurring before baseline data collection with 19 participants (24.4%) who dropped out. An additional eight participants (10%) withdrew before the second assessment/3-month, and three participants (4%) before the third assessment/6-month. At study completion, 26 participants remained in the wait-list control group and 22 in the intervention group. Reasons for dropouts for all timepoints included, lost to follow up (n=19), language barrier (n=1), no longer interested in the study (n=1), no time to complete the study (n=2), fraudulent/attempted multiple enrollments (n=3), withdrew (n=2), removed from study (n=4). Following allocation, about half (n=14, 45%) of participants in the immediate intervention arm actively engaged with content in the VE. Within the intervention arm, 14 participants (45%) logged into the VE at least once. "

13b-i) Attrition diagram

Strongly recommended: An attrition diagram (e.g., proportion of participants still logging in or using the intervention/comparator in each group plotted over time, similar to a survival curve) or other figures or tables demonstrating usage/dose/engagement.

	1	2	3	4	5	
subitem not at all important	○	○	○	○	○	essential

Your response is too large. Try shortening some answers.

Does your paper address subitem 13b-i?

Copy and paste relevant sections from the manuscript or cite the figure number if applicable (include quotes in quotation marks "like this" to indicate direct quotes from your manuscript), or elaborate on this item by providing additional information not in the ms, or briefly explain why the item is not applicable/relevant for your study

Your answer

14a) Dates defining the periods of recruitment and follow-up

Does your paper address CONSORT subitem 14a? *

Copy and paste relevant sections from the manuscript (include quotes in quotation marks "like this" to indicate direct quotes from your manuscript), or elaborate on this item by providing additional information not in the ms, or briefly explain why the item is not applicable/relevant for your study

"Between July 2023 and December 2024, individuals were screened to participate and their duration went to six months from enrollment date."

14a-i) Indicate if critical "secular events" fell into the study period

Indicate if critical "secular events" fell into the study period, e.g., significant changes in Internet resources available or "changes in computer hardware or Internet delivery resources"

	1	2	3	4	5	
subitem not at all important	○	○	○	○	○	essential

Your response is too large. Try shortening some answers.

Does your paper address subitem 14a-i?

Copy and paste relevant sections from the manuscript (include quotes in quotation marks "like this" to indicate direct quotes from your manuscript), or elaborate on this item by providing additional information not in the ms, or briefly explain why the item is not applicable/relevant for your study

Your answer

14b) Why the trial ended or was stopped (early)

Does your paper address CONSORT subitem 14b? *

Copy and paste relevant sections from the manuscript (include quotes in quotation marks "like this" to indicate direct quotes from your manuscript), or elaborate on this item by providing additional information not in the ms, or briefly explain why the item is not applicable/relevant for your study

The trial was not ended early.

15) A table showing baseline demographic and clinical characteristics for each group

NPT: When applicable, a description of care providers (case volume, qualification, expertise, etc.) and centers (volume) in each group

Your response is too large. Try shortening some answers.

Does your paper address CONSORT subitem 15? *

Copy and paste relevant sections from the manuscript (include quotes in quotation marks "like this" to indicate direct quotes from your manuscript), or elaborate on this item by providing additional information not in the ms, or briefly explain why the item is not applicable/relevant for your study

"Table 1 provides a summary of participant demographic characteristics. The mean age of participants was 41.8 years (SD = 10.4). The wait-list control group had a mean age of 42.6 years (SD = 11.2), while the intervention group had a mean age of 41.1 years (SD = 9.7). Participants had been living with HIV for an average of 15.9 years (SD = 9.3). The racial composition was as follows: 62.5% Black, 26.8% Hispanic/Latine, and approximately 1.8% Asian and 1.8% Middle Eastern. A total of 62.1% of participants were single and 22.4% were married. Participants reported a range of educational backgrounds, with most completing some college at 29.3%, and either have a Bachelor's or Master's degrees at 24.1% each. Employment status varied, with 48.3% employed and 36.2% unemployed or working seasonally."

15-i) Report demographics associated with digital divide issues

In ehealth trials it is particularly important to report demographics associated with digital divide issues, such as age, education, gender, social-economic status, computer/Internet/ehealth literacy of the participants, if known.

	1	2	3	4	5	
subitem not at all important	(●)	( )	( )	( )	( )	essential

Clear selection

Your response is too large. Try shortening some answers.

Does your paper address subitem 15-i? *

Copy and paste relevant sections from the manuscript (include quotes in quotation marks "like this" to indicate direct quotes from your manuscript), or elaborate on this item by providing additional information not in the ms, or briefly explain why the item is not applicable/relevant for your study

"Figure 5 CONSORT diagram provides summary of feasibility results. A total of 170 individuals were screened and assessed for eligibility, of which 92 were excluded (n=46 did not meet inclusion criteria, and n=46 declined to participate). In the RCT, 78 of the targeted 80 participants were enrolled (97.5%), demonstrating successful recruitment. Of these 78 participants, 40 entered the intervention group and 38 entered the wait-list control group.

Acceptability:
Table 2 provides a summary of acceptability results. Participants logged a total of 110 sessions, averaging 1 hour and 50 minutes each. Quests were accessed 14 times with the most frequently visited quests located in the fitness district (64.0%), followed by the grocery district (14.0%), and the pharmacy, bookstore, and outlet districts (7.0% each). The most engaged content were nutrition (146 engagements), oral health (138 engagements), fitness tips and videos (82 engagements), and relaxation techniques (75 engagements)."

16) For each group, number of participants (denominator) included in each analysis and whether the analysis was by original assigned groups

16-i) Report multiple "denominators" and provide definitions

Report multiple "denominators" and provide definitions: Report N's (and effect sizes) "across a range of study participation [and use] thresholds" [1], e.g., N exposed, N consented, N used more than x times, N used more than y weeks, N participants "used" the intervention/comparator at specific pre-defined time points of interest (in absolute and relative numbers per group). Always clearly define "use" of the intervention.

	1	2	3	4	5	
subitem not at all important	○	○	○	○	○	essential

Your response is too large. Try shortening some answers.

Does your paper address subitem 16-i? *

Copy and paste relevant sections from the manuscript (include quotes in quotation marks "like this" to indicate direct quotes from your manuscript), or elaborate on this item by providing additional information not in the ms, or briefly explain why the item is not applicable/relevant for your study

Participants logged a total of 110 sessions, averaging 1 hour and 50 minutes each. Quests were accessed 14 times with the most frequently visited quests located in the fitness district (64.0%), followed by the grocery district (14.0%), and the pharmacy, bookstore, and outlet districts (7.0% each). The most engaged content were nutrition (146 engagements), oral health (138 engagements), fitness tips and videos (82 engagements), and relaxation techniques (75 engagements)." "The exploratory effect sizes observed in this study are consistent with, and in some cases exceed, those reported in comparable digital health interventions. For dietary outcomes, medium effect sizes for vegetable consumption (d=0.59 at 3-months to 0.66 at 6-months) and whole grain intake (d=0.41 at 3-months to 0.46 at 6-months) compare favorably to meta-analytic findings from technology-based dietary interventions, which typically report small-to-medium effects.59,60 The sustained improvement at 6-months suggests the VE format may support durable behavior change, though the attenuation of sweets reduction (d=-0.65 at 3-months to 0.01 at 6-months) indicates that some dietary behaviors may require ongoing reinforcement. The improvement in vigorous physical activity (d=0.38 at 3-months to d=0.57 at 6-months) is noteworthy and comparable to a meta-analysis finding61 of physical activity interventions showing larger effects at 6-9 months; though the extent that these interventions were delivered virtually or digitally were not specified.61 The gamified, self-directed nature of the VE may have promoted autonomous motivation, which has been posited to impact sustained physical activity adherence in self-determination theory frameworks.62,63 We also noted that more participants with higher BMI were retained in the intervention group compared to the control group; while it is plausible that the virtual format reduced weight-related stigma, offered an engaging self-directed experience, or was perceived as more personally relevant by this subgroup, reasons for this differential retention warrant further study. Moreover, while any improvement emerged from an intervention not targeting mental health and illness perceptions directly is notable, the small effect sizes for depressive symptoms (d=0.22) and illness perceptions (d=0.23 at 3-months and 0.33) from a low-intensity, self-directed VE intervention warrant further investigation in a fully powered trial. These preliminary shifts in these domains may be precursors to sustained behavioral change, though larger samples are needed to confirm these preliminary effect size patterns. "

Your response is too large. Try shortening some answers.

16-ii) Primary analysis should be intent-to-treat

Primary analysis should be intent-to-treat, secondary analyses could include comparing only “users”, with the appropriate caveats that this is no longer a randomized sample (see 18-i).

	1	2	3	4	5	
subitem not at all important	○	○	○	○	○	essential

Does your paper address subitem 16-ii?

Copy and paste relevant sections from the manuscript (include quotes in quotation marks "like this" to indicate direct quotes from your manuscript), or elaborate on this item by providing additional information not in the ms, or briefly explain why the item is not applicable/relevant for your study

Your answer

17a) For each primary and secondary outcome, results for each group, and the estimated effect size and its precision (such as 95% confidence interval)

Does your paper address CONSORT subitem 17a? *

Copy and paste relevant sections from the manuscript (include quotes in quotation marks "like this" to indicate direct quotes from your manuscript), or elaborate on this item by providing additional information not in the ms, or briefly explain why the item is not applicable/relevant for your study

"Table 2 provides a summary of acceptability results. Participants logged a total of 110 sessions, averaging 1 hour and 50 minutes each. Quests were accessed 14 times with the most frequently visited quests located in the fitness district (64.0%), followed by the grocery district (14.0%), and the pharmacy, bookstore, and outlet districts (7.0% each). The most engaged content were nutrition (146 engagements), oral health (138 engagements), fitness tips and videos (82 engagements), and relaxation techniques (75 engagements). "

Your response is too large. Try shortening some answers.

17a-i) Presentation of process outcomes such as metrics of use and intensity of use

In addition to primary/secondary (clinical) outcomes, the presentation of process outcomes such as metrics of use and intensity of use (dose, exposure) and their operational definitions is critical. This does not only refer to metrics of attrition (13-b) (often a binary variable), but also to more continuous exposure metrics such as "average session length". These must be accompanied by a technical description how a metric like a "session" is defined (e.g., timeout after idle time) [1] (report under item 6a).

	1	2	3	4	5	
subitem not at all important	○	○	○	○	○	essential

Does your paper address subitem 17a-i?

Copy and paste relevant sections from the manuscript (include quotes in quotation marks "like this" to indicate direct quotes from your manuscript), or elaborate on this item by providing additional information not in the ms, or briefly explain why the item is not applicable/relevant for your study

Your answer

17b) For binary outcomes, presentation of both absolute and relative effect sizes is recommended

Does your paper address CONSORT subitem 17b? *

Copy and paste relevant sections from the manuscript (include quotes in quotation marks "like this" to indicate direct quotes from your manuscript), or elaborate on this item by providing additional information not in the ms, or briefly explain why the item is not applicable/relevant for your study

We did not report on this

Your response is too large. Try shortening some answers.

18) Results of any other analyses performed, including subgroup analyses and adjusted analyses, distinguishing pre-specified from exploratory

Does your paper address CONSORT subitem 18? *

Copy and paste relevant sections from the manuscript (include quotes in quotation marks "like this" to indicate direct quotes from your manuscript), or elaborate on this item by providing additional information not in the ms, or briefly explain why the item is not applicable/relevant for your study

We did not perform any ancillary analyses

18-i) Subgroup analysis of comparing only users

A subgroup analysis of comparing only users is not uncommon in ehealth trials, but if done, it must be stressed that this is a self-selected sample and no longer an unbiased sample from a randomized trial (see 16-iii).

	1	2	3	4	5	
subitem not at all important	○	○	○	○	○	essential

Does your paper address subitem 18-i?

Copy and paste relevant sections from the manuscript (include quotes in quotation marks "like this" to indicate direct quotes from your manuscript), or elaborate on this item by providing additional information not in the ms, or briefly explain why the item is not applicable/relevant for your study

Your answer

19) All important harms or unintended effects in each group
(for specific guidance see CONSORT for harms)

Your response is too large. Try shortening some answers.

Does your paper address CONSORT subitem 19? *

Copy and paste relevant sections from the manuscript (include quotes in quotation marks "like this" to indicate direct quotes from your manuscript), or elaborate on this item by providing additional information not in the ms, or briefly explain why the item is not applicable/relevant for your study

"Of note, there were one or two instances of individuals attempting to enroll multiple times. The study coordinator employed a rigorous, multi-step verification process that included phone screenings, email confirmations, checks for geographic inconsistencies, and IP address verification along with location screening. This approach allowed her to detect discrepancies, such as registrations emanating from different continents and participants utilizing virtual private networks (VPNs) to mask their actual locations. The challenge of multiple enrollment attempts has become increasingly prevalent in remote research settings, and no evidence-based strategies currently exist.59,60 However, our comprehensive approach effectively mitigated this risk, resulting in no duplicate enrollments." "To enhance retention various strategies were implemented, such as maintaining regular contact through telephone calls and emails, disseminating monthly newsletters celebrating relevant events such as Pride and paying homage to the legacy activists who paved the way (e.g., Marsha P. Johnson, Sylvia Rivera, and countless others) towards visibility, representation, and the right to exist. These communications offered community connection between the team and the participants. The positive feedback received through “thank-you” emails and phone communications with the study coordinator suggested that maintaining cultural relevance and community acknowledgment, which are key community-based research principles,58 is particularly important for engaging underrepresented groups in longitudinal research. "

19-i) Include privacy breaches, technical problems

Include privacy breaches, technical problems. This does not only include physical “harm” to participants, but also incidents such as perceived or real privacy breaches [1], technical problems, and other unexpected/unintended incidents. “Unintended effects” also includes unintended positive effects [2].

	1	2	3	4	5	
subitem not at all important	○	○	○	○	○	essential

Your response is too large. Try shortening some answers.

Does your paper address subitem 19-i?

Copy and paste relevant sections from the manuscript (include quotes in quotation marks "like this" to indicate direct quotes from your manuscript), or elaborate on this item by providing additional information not in the ms, or briefly explain why the item is not applicable/relevant for your study

Your answer

19-ii) Include qualitative feedback from participants or observations from staff/researchers

Include qualitative feedback from participants or observations from staff/researchers, if available, on strengths and shortcomings of the application, especially if they point to unintended/unexpected effects or uses. This includes (if available) reasons for why people did or did not use the application as intended by the developers.

	1	2	3	4	5	
subitem not at all important	○	○	○	○	○	essential

Does your paper address subitem 19-ii?

Copy and paste relevant sections from the manuscript (include quotes in quotation marks "like this" to indicate direct quotes from your manuscript), or elaborate on this item by providing additional information not in the ms, or briefly explain why the item is not applicable/relevant for your study

Your answer

DISCUSSION

Your response is too large. Try shortening some answers.

22) Interpretation consistent with results, balancing benefits and harms, and considering other relevant evidence

NPT: In addition, take into account the choice of the comparator, lack of or partial blinding, and unequal expertise of care providers or centers in each group

22-i) Restate study questions and summarize the answers suggested by the data, starting with primary outcomes and process outcomes (use)

Restate study questions and summarize the answers suggested by the data, starting with primary outcomes and process outcomes (use).

	1	2	3	4	5	
subitem not at all important	○	○	○	○	◉	essential

Clear selection

Your response is too large. Try shortening some answers.

Does your paper address subitem 22-i? *

Copy and paste relevant sections from the manuscript (include quotes in quotation marks "like this" to indicate direct quotes from your manuscript), or elaborate on this item by providing additional information not in the ms, or briefly explain why the item is not applicable/relevant for your study

The findings of the LEARN RCT demonstrated feasibility, as we enrolled 78 participants, achieving 98% of the targeted sample size. This outcome is noteworthy given that approximately 25% of randomized controlled trials fail to meet recruitment goals, even with extended trial periods.53 The high enrollment rate reflects the effectiveness of multiple recruitment strategies employed, including community partnerships, digital outreach through MyChart messaging campaigns, research listservs, and targeted communications. Retention presented challenges typically associated with tech-driven longitudinal research, with a final retention rate of 61.5% at the 6-month assessment. While modest, our retention outcome exceeded the typical rates observed in remote digital health initiatives, which peak around 56%.54-56 Attrition primarily occurred prior to baseline data collection, with 19 participants (24%) withdrawing after verbal consent, consistent with research indicating that more than half of discontinuations in digital health studies occur within the first week.57 To enhance retention various strategies were implemented, such as maintaining regular contact through telephone calls and emails, disseminating monthly newsletters celebrating relevant events such as Pride and paying homage to the legacy activists who paved the way (e.g., Marsha P. Johnson, Sylvia Rivera, and countless others) towards visibility, representation, and the right to exist. These communications offered community connection between the team and the participants. The positive feedback received through “thank-you” emails and phone communications with the study coordinator suggested that maintaining cultural relevance and community acknowledgment, which are key community-based research principles,58 is particularly important for engaging underrepresented groups in longitudinal research.

22-ii) Highlight unanswered new questions, suggest future research

Highlight unanswered new questions, suggest future research.

	1	2	3	4	5	
subitem not at all important	○	○	○	○	○	essential

Your response is too large. Try shortening some answers.

Does your paper address subitem 22-ii?

Copy and paste relevant sections from the manuscript (include quotes in quotation marks "like this" to indicate direct quotes from your manuscript), or elaborate on this item by providing additional information not in the ms, or briefly explain why the item is not applicable/relevant for your study

Your answer

20) Trial limitations, addressing sources of potential bias, imprecision, and, if relevant, multiplicity of analyses

20-i) Typical limitations in ehealth trials

Typical limitations in ehealth trials: Participants in ehealth trials are rarely blinded. Ehealth trials often look at a multiplicity of outcomes, increasing risk for a Type I error. Discuss biases due to non-use of the intervention/usability issues, biases through informed consent procedures, unexpected events.

	1	2	3	4	5	
subitem not at all important	○	○	○	○	◉	essential

Clear selection

Your response is too large. Try shortening some answers.

Does your paper address subitem 20-i? *

Copy and paste relevant sections from the manuscript (include quotes in quotation marks "like this" to indicate direct quotes from your manuscript), or elaborate on this item by providing additional information not in the ms, or briefly explain why the item is not applicable/relevant for your study

"The study has several limitations that warrant consideration. First, the sample was limited to men with HIV, which may affect generalizability to other populations and chronic conditions. Second, technical challenges with the platform due to its age (e.g., freezing, audio problems, and installation difficulties) likely constrained engagement, though the study team worked closely with developers to provide ongoing troubleshooting support. Third, given our study's retention rate and engagement which diminished over time, future iterations could mitigate this by refining screening and inclusion criteria to identify characteristics of participants who were not retained and/or developing a new platform and will incorporate additional strategies to maintain participant engagement, such as personalized content, regular updates, and incentives for continued participation. Fourth, the exploratory nature of secondary outcomes means these findings were not powered to detect significant changes in among the health indicators explored as secondary outcomes of this study. Future research should build on these exploratory findings to develop and test more targeted interventions aimed at improving specific health behaviors and outcomes."

21) Generalisability (external validity, applicability) of the trial findings

NPT: External validity of the trial findings according to the intervention, comparators, patients, and care providers or centers involved in the trial

21-i) Generalizability to other populations

Generalizability to other populations: In particular, discuss generalizability to a general Internet population, outside of a RCT setting, and general patient population, including applicability of the study results for other organizations

	1	2	3	4	5	
subitem not at all important	○	○	○	○	○	essential

Your response is too large. Try shortening some answers.

Does your paper address subitem 21-i?

Copy and paste relevant sections from the manuscript (include quotes in quotation marks "like this" to indicate direct quotes from your manuscript), or elaborate on this item by providing additional information not in the ms, or briefly explain why the item is not applicable/relevant for your study

Your answer

21-ii) Discuss if there were elements in the RCT that would be different in a routine application setting

Discuss if there were elements in the RCT that would be different in a routine application setting (e.g., prompts/reminders, more human involvement, training sessions or other co-interventions) and what impact the omission of these elements could have on use, adoption, or outcomes if the intervention is applied outside of a RCT setting.

	1	2	3	4	5	
subitem not at all important	○	○	○	○	○	essential

Does your paper address subitem 21-ii?

Copy and paste relevant sections from the manuscript (include quotes in quotation marks "like this" to indicate direct quotes from your manuscript), or elaborate on this item by providing additional information not in the ms, or briefly explain why the item is not applicable/relevant for your study

Your answer

OTHER INFORMATION

23) Registration number and name of trial registry

Your response is too large. Try shortening some answers.

Does your paper address CONSORT subitem 23? *

Copy and paste relevant sections from the manuscript (include quotes in quotation marks "like this" to indicate direct quotes from your manuscript), or elaborate on this item by providing additional information not in the ms, or briefly explain why the item is not applicable/relevant for your study

ClinicalTrials.gov NCT05242952

24) Where the full trial protocol can be accessed, if available

Does your paper address CONSORT subitem 24? *

Cite a Multimedia Appendix, other reference, or copy and paste relevant sections from the manuscript (include quotes in quotation marks "like this" to indicate direct quotes from your manuscript), or elaborate on this item by providing additional information not in the ms, or briefly explain why the item is not applicable/relevant for your study

https://www.researchprotocols.org/2022/5/e38348

25) Sources of funding and other support (such as supply of drugs), role of funders

Does your paper address CONSORT subitem 25? *

Copy and paste relevant sections from the manuscript (include quotes in quotation marks "like this" to indicate direct quotes from your manuscript), or elaborate on this item by providing additional information not in the ms, or briefly explain why the item is not applicable/relevant for your study

National Heart, Lung, and Blood Institute (K01HL145580)

X27) Conflicts of Interest (not a CONSORT item)

Your response is too large. Try shortening some answers.

X27-i) State the relation of the study team towards the system being evaluated

In addition to the usual declaration of interests (financial or otherwise), also state the relation of the study team towards the system being evaluated, i.e., state if the authors/evaluators are distinct from or identical with the developers/sponsors of the intervention.

	1	2	3	4	5	
subitem not at all important	○	○	○	○	○	essential

Does your paper address subitem X27-i?

Copy and paste relevant sections from the manuscript (include quotes in quotation marks "like this" to indicate direct quotes from your manuscript), or elaborate on this item by providing additional information not in the ms, or briefly explain why the item is not applicable/relevant for your study

Your answer

About the CONSORT EHEALTH checklist

As a result of using this checklist, did you make changes in your manuscript? *

- ○ yes, major changes
- ○ yes, minor changes
- ● no

Your response is too large. Try shortening some answers.

What were the most important changes you made as a result of using this checklist?

we have included the majority of the checklist during drafting of this manuscript. It was very helpful for the drafting and including items.

How much time did you spend on going through the checklist INCLUDING making changes in your manuscript *

This took multiple days and hours to complete

As a result of using this checklist, do you think your manuscript has improved? *

- (x) yes
- ( ) no
- ( ) Other:

Would you like to become involved in the CONSORT EHEALTH group?

This would involve for example becoming involved in participating in a workshop and writing an "Explanation and Elaboration" document

- ( ) yes
- ( ) no
- ( ) Other:

Your response is too large. Try shortening some answers.

Any other comments or questions on CONSORT EHEALTH

Your answer

STOP - Save this form as PDF before you click submit

To generate a record that you filled in this form, we recommend to generate a PDF of this page (on a Mac, simply select "print" and then select "print as PDF") before you submit it.

When you submit your (revised) paper to JMIR, please upload the PDF as supplementary file.

Don't worry if some text in the textboxes is cut off, as we still have the complete information in our database. Thank you!

Final step: Click submit !

Click submit so we have your answers in our database!

Submit

Clear form

Never submit passwords through Google Forms.

This content is neither created nor endorsed by Google. - Terms of Service - Privacy Policy

Does this form look suspicious? Report

Google Forms

Your response is too large. Try shortening some answers.

Your response is too large. Try shortening some answers.
