# Supplementary material for: A Web-Based Virtual Environment Behavioral Intervention as Cardiovascular Disease and Metabolic Disease Prevention Education in Persons With HIV: Evaluation of the LEARN Randomized Controlled Trial
Source: J Med Internet Res. 2026 Jun 15;28:e91145. doi: 10.2196/91145 (PMC13316017; doi:10.2196/91145)
Supplement: Multimedia Appendix 2 [file jmir_v28i1e91145_app2.docx]

|  | Total |
| --- | --- |
| **Acceptability:** |  |
| Total sessions logged | 110 |
| Average session duration, in minutes | 110 |
| Distinct educational quests accessed | 14 |
| Most visited districts | Fitness: 64.0%  Grocery: 14.0%  Pharmacy: 7.0%  Bookstore: 7.0%  Outlet: 7.0% |
| Most engaged modules | Nutrition: k=146  Oral health: k=138  Fitness tips & videos: k=82  Relaxation techniques: k=75  Health and Beauty: k=73  Skin Care: k=71  Topics on Diabetes: k=66  Health Supplies: k=62  Medication: k=55  Complications: k=41  Supplements: k=41  Fast Food: k=39  Fitness Videos: k=37  Snacks: k=30  Chain Restaurants: k=21  Prepared Foods: k=20  Beverages: k=18  Frozen Food: k=17  Groceries: k=17  Produce: k=16  Dairy: k=12  Meat & Seafood: k=11  Health Education & Technology Support: k=8  Health Living: k=7  Bakery: k=3  Packaged: k=2 |
